# Genome-wide identification and unveiling the role of MAP kinase cascade genes involved in sugarcane response to abiotic stressors

**DOI:** 10.1186/s12870-025-06490-1

**Published:** 2025-04-16

**Authors:** Ahmad Ali, Xue-Ting Zhao, Ji-Shan Lin, Ting-Ting Zhao, Cui-Lian Feng, Ling Li, Rui-Jie Wu, Qi-Xing Huang, Hong-Bo Liu, Jun-Gang Wang

**Affiliations:** 1 National Key Laboratory for Tropical Crop Breeding, Institute of Tropical Bioscience and Biotechnology, Chinese Academy of Tropical Agricultural Sciences, Haikou, 571101 China; 2 Key Laboratory of Biology and Genetic Resources of Tropical Crops/Key Laboratory for Biology and Genetic Resources of Tropical Crops of Hainan Province, Hainan Institute for Tropical Agricultural Resources, Sanya Institute of Chinese Academy of Tropical Agricultural Sciences, Sanya, 572024 China; 3 National Key Laboratory for Tropical Crop Breeding, Sugarcane Research Institute, Yunnan Key Laboratory of Sugarcane Genetic Improvement, Yunnan Academy of Agricultural Sciences, Kaiyuan, 661699 China

**Keywords:** MAPK cascade, Genome-wide, Sugarcane, Expression patterns, Abiotic stress responses

## Abstract

**Background:**

The MAP Kinase cascade system is a conserved signaling mechanism essential for plant development, growth, and stress tolerance. Thus far, genes from the MAPK cascade have been identified in several plant species but remain uncharacterized in the polyploid *Saccharum* spp. Hybrid R570 genome.

**Results:**

This study identified 89 *ScMAPK*, 24 *ScMAPKK*, and 107 *ScMAPKKK* genes through genome-wide analysis. Phylogenetic classification revealed that four subgroups were present in each *ScMAPK* and *ScMAPKK* family, and three sub-families (ZIK-like, RAF-like, and MEKK-like) presented in the *ScMAPKKK* family. Conserved motif and gene structure analysis supported the evolutionary relationships of the three families inferred from the phylogenetic analysis. All of the *ScMAPK*, *ScMAPKK* and *ScMAPKKK* genes were mapped on four scaffolds (Scaffold_88/89/91/92) and nine chromosomes (1–8, 10). Collinearity and gene duplication analysis identified 169 pairs of allelic and non-allelic segmentally duplicated MAPK cascade genes, contributing to their expansion. Additionally, 13 putative ‘ss-miRNAs’ were predicted to target 87 MAPK cascade genes, with ‘ssp-miR168a’ alone regulating 45 genes. qRT-PCR analysis revealed differential gene expression under abiotic stressors. *ScMAPK07*, *ScMAPK66*, and *ScRAF43* were down-regulated and acted as negative regulators. Conversely, *ScMAPKK13*, *ScRAF10*, and *ScZIK18* were up-regulated at specific time points under drought, with *ScZIK18* exhibiting strong defense. Under NaCl stress, most genes were down-regulated, except for slight increases in *ScZIK18* and *ScMAPKK13*, suggesting a positive role in salt stress response. Under CaCl_2_ stress, five genes were significantly down-regulated, while *ScRAF43* remained unchanged, reflecting their negative roles in stress adaptation and resource conservation.

**Conclusion:**

This study provides insights into MAPK cascade gene evolution and function in sugarcane, highlighting distinct regulatory roles in abiotic stress responses. Interestingly, some genes acted as negative regulators, serving as a mechanism to balance stress responses and prevent overactivation. In contrast, others contributed to defense mechanisms, offering potential targets for stress resilience improvement.

**Clinical Trail Number:**

This study contains no clinical trials. Not applicable.

**Supplementary Information:**

The online version contains supplementary material available at 10.1186/s12870-025-06490-1.

## Introduction

The mitogen-activated protein kinase (MAPK) cascade is a conserved signaling module present in all eukaryotic organisms. It transforms signals from receptors or sensors into cellular responses, playing a key role in regulating or controlling plant growth and development [1]. This pathway typically involves three sequentially acting protein kinases, including MAP kinases (MAPKs), MAP kinase kinases (MAPKKs) and MAP kinase kinase kinases (MAPKKKs) [1, 2]. MAPKKKs, as serine/threonine (Ser/Thr) protein kinases, activate MAPKKs by phosphorylation of two (Ser/Thr) residues within the S/T-5x-S/T motif. MAPKKs, which function as bispecific kinases, activate MAPKs by phosphorylating Ser/Thr residues within the T-[D/E]-Y motif. The activated MAPKs, in turn, regulate downstream targets, including transcription factors (TFs), protein kinases, and other signaling components, to orchestrate several biological processes such as metabolism, defense responses, and development [1–3]. To date, MAPK cascade genes have been identified in numerous plant species, including *Saccharum* spp. Hybrid R570 monoploid genome [2], *Zea mays* [4], *Fagopyrum tataricum* [5, 6], *Solanum tuberosum* L [7]., *Setaria italica* [3], and *Camellia oleifera* [8]. These discoveries provide valuable insights and resources for advancing research on the MAPK signaling pathway.

The MAPK cascade is an extremely conserved signaling pathway in higher plants, crucial for cell division, development and mediating responses to abiotic stressors such as heavy metal tolerance, drought, calcium, salinity, low temperature and cold [1, 9]. Studies showed that plants undergo physiological, biochemical, or molecular alterations when subjected to abiotic stressors. These responses are coordinated through multilevel regulatory processes, including sophisticated signaling networks produced by plants that facilitate a particular response in plants [10]. The MEKK1-MKK1/MKK-MPK4/6-MKS1/WRKY33 regulates innate immunity and mediated response to cold and salt stress response [11, 12]. In addition, a study showed that low temperature triggers Ca^2+^-mediated signaling, activating *CRLK1*, which then phosphorylates and activates *MEKK1*, an upstream factor in the MAPK cascade. Activation of *MEKK1* by *MKK2* leads to the *MPK4* and *MPK6* phosphorylation. These findings suggested that Ca^2+^ signaling therefore plays a crucial part in MAPK-mediated cold stress response in plants [12]. In cotton, *GhMPK17* and *GhMKK3* were revealed to contribute to the plant response to high salt stress, and abscisic acid (ABA) signaling, while promoting drought resistance through stomatal response and root growth [13, 14]. Similarly, *GhRAF4* and *GhMEKK12* silencing reduces resilience to drought stress in cotton plants [15], whereas *OsMAPKKK63* interacts with *OsMKK1* and *OsMKK6* to respond to salinity stress and regulate seed dormancy [16]. In addition, a study by Verma and colleagues showed that the *MKK3*-*MPK6* cascade regulates *MYC2*, a *bHLH* TF, which repressed *P5CS1* gene expression in proline biosynthesis, thereby negatively regulating salt stress response [17]. Recently, Yan and colleagues revealed that MPK3 and MPK6 interact and further phosphorylate crucial cytokinin signaling components (*ARR1/10/12*) to boost their degradation, increasing salt tolerance to *mpk3* and *mpk6* double mutants in Arabidopsis [18].

Sugarcane (*Saccharum* spp.) accounts for 80% of global sugar production and 90% of total sugar yield in China. It also holds significant potential for bioethanol production [19, 20]. The genome of modern sugarcane cultivars is complex, with high levels of polyploidy and aneuploidy (2n = 100–130). Of this complexity, 80% originates from *S. officinarum* (2n = 80) and 10–15% from *S. spontaneum* (2n = 40–128) [21]. *S. officinarum* offers traits related to elevated sugar content and extra hardness, while *S. spontaneum* offers genes related to disease resistance and ratooning ability [22–24]. Notably, the *Saccharum* spp. Hybrid R570 cultivar (2n = 114) have monoploid and polyploid genomes that offer valued resources to uncover several gene families with divers functions [23, 25]. The monoploid R570 genome was assembled using 4,660 BAC clones (~ 100 kb each) to represent gene-rich regions of the sorghum genome, resulting in a 382-Mb assembly with 25,316 predicted protein-coding genes across 10 pseudo-chromosomes [23]. In contrast, the highly polyploid R570 genome, with 114 chromosomes (~ 20% from *S. spontaneum*), was assembled, which consists of a 5.04 Gb primary genome with 67 pseudo-chromosomes containing 194,593 predicted genes and a 3.7 Gb alternate assembly of highly similar contigs [25]. Previously, numerous *ShMAPK*, *ShMAPKK*, and *ShMAPKKK* family genes were identified in the monoploid assembly of the R570 cultivar, and their transcript levels were observed in response to biotic and abiotic stressors [2]. However, no reports are available on the identification of MAPK cascade genes in the polyploid assembly of the R570 hybrid cultivar and their responses to abiotic stresses in sugarcane. This study identified and classified *ScMAPK*, *ScMAPKK*, and *ScMAPKKK* gene families in the polyploid R570 hybrid cultivar genome and investigated their expression patterns in response to sodium chloride (NaCl), polyethylene glycol (PEG6000) and calcium chloride (CaCl_2_) stress in sugarcane. This study will provide a foundation for understanding the evolution of the MAPK cascade family genes in sugarcane, offering valuable insights for further research.

## Materials and methods

### Sequence retrieval of MAPK cascade genes in *Saccharum* spp. Hybrid R570 genome

The genomic sequences for the polyploid *Saccharum* spp. Hybrid R570 [25] was downloaded from the sugarcane genome database web server (https://sugarcane-genome.cirad.fr/, retrieved on April 2024). The BLASTp algorithm was used to confirm the genes encoding *ScMAPKs*,* ScMAPKKs*, and *ScMAPKKKs* from the R570 genome to ensure the maximum number of gene retrievals. The 37 *Saccharum* MAPK cascades gene sequences [2] were retrieved from the *Saccharum* spp. Hybrid R570 cultivar monoploid assembly. These sequences were further used as bait in a BLASTp search (similarity percentage: ≥ 90%, e-value: ≤ 1 × 10^− 4^ and high bit score: 746–1540). The Pfam online website (available at http://xfam.org/, accessed on 2 May 2024) was searched for the HMM (Hidden Markov Model) file associated with MAP kinase domains (STKc and PKc). HMMER (version 2.41.2) was used to retrieve MAPK cascade genes from the polyploid R570 hybrid cultivar [25] genome database. False sequences were discarded manually. The final MAPK cascade sequences were confirmed by identifying their functional domains via the CDD-batch (Conserved Domain Database) tool available on NCBI (https://www.ncbi.nlm.nih.gov, accessed on 3 May 2024), Pfam online web database (available at http://xfam.org/, accessed on 3 May 2024), and SMART (available at http://smart.emblheidelberg.de/, accessed on 3 May 2024).

### Physiochemical parameters prediction and orthologous gene clusters identification

The orthologous MAPK, MAPKK, and MAPKKK protein sequences in *Saccharum* spp. Hybrid R570, *Saccharum* spp. Hybrid SP80-3280 [26], *S. spontaneum* AP85-384 [24], and *S. spontaneum* Np-X [27] were discovered through Orthovenn3 database (available at https://orthovenn3.bioinfotoolkits.net/, accessed on 21 May 2024). The ProtParam ExPASy online web server (available at https://web.expasy.org/, accessed on 15 May 2024) and Tbtools-II software were operated to determine the physiochemical parameters of these MAPK cascades proteins, for example, molecular weights (Mw), number of amino acids (aa), isoelectric points (pI), instability index (II), and GRAVY. The WoLF-PSORT online web portal (available at https://wolfpsort.hgc.jp/, accessed on 15 May 2024) was employed to figure out sub-cellular localization (SL).

### Phylogenetic and multiple sequence analysis

The protein sequences of MAP Kinase cascade genes of *Z. mays* (Zm) (http://www.maizegdb.org/, accessed on 27 May 2024), *A. thaliana* (At) (available at https://www.arabidopsis.org, accessed on 27 May 2024), *Jatropha curcas* (Jc) (available at http://www.kazusa.or.jp/, accessed on 27 May 2024), and *Gossypium raimondii* (Gr) (available at http://www.phytozome.net/, accessed on 27 May 2024) genome databases. Phylogenetic analysis was conducted via the Nj method in MEGA11 software (V11.0.1). Statistical reliability was assessed through bootstrap analysis with 1000 replicates. Multiple sequence alignments of 220 MAP Kinase cascade proteins were performed by the ClustalX2 algorithm implemented in the MEGA11 program with default parameters [28].

### Gene structure, conserved motif and *cis*-regulatory elements study

Data for the three gene families (*ScMAPK*, *ScMAPKK*, and *ScMAPKKK*) were retrieved from the R570 hybrid genome database [25]. The data including information on accession number, chromosomal location (CL), and exon-intron structures were extracted. The CDS and gene sequences of the known MAP Kinase cascade genes were used to construct the gene structure, including exon-intron distribution. This analysis was conducted using the GSDS online web tool (available at http://gsds.gao-lab.org/, accessed on 29 May 2024) and TBtools-II (Toolbox for Biologists v2.131) software [29]. Conserved motif analysis of all the identified MAPK cascade protein sequences was performed using MEME Suite online web server (v5.5.7) (available at https://meme-suite.org/meme/, accessed on 29 May 2024) to detect the occurrence of 10 predefined motifs in the input sequences. The search was restricted to allow zero or one occurrence per sequence, ensuring each motif was present at most once in any given sequence. The TBtools-II software v2.131 was used to visualize these motif patterns. *Cis*-acting elements analysis was conducted by scanning the 2000-kb (upstream of the start codon) promoter sequences of all MAPK cascades genes at PlantCARE online web tool (available at https://bioinformatics.psb.ugent.be/webtools/plantcare/, accessed on 30 May 2024) then construct by TBtools-II software.

### Chromosomal localization, gene duplication and synteny analysis

The CL of *ScMAPK*, *ScMAPKK*, and *ScMAPKKK* gene families were obtained from the general feature format 3 (gff3) file, and the chromosome number, start and end position of each gene were extracted and visualized using TBtools-II software [29]. Comparative synteny analysis was conducted to examine the evolutionary conservation of genomes among the polyploid *Saccharum* spp. Hybrid R570 and the two *S. spontaneum* clones (Np-X, and AP85-441) whole genomes. All genomic files were scanned using MCScanX (Super-fast) toolkit in TBtools-II software, and the resulting files were used to generate multiple synteny maps. The gene duplicated events were investigated by running the super-fast MCScanX toolkit on each *Saccharum* genome.

### Prediction of MiRNAs targeting *Saccharum* MAPK cascade genes

To understand the underlying regulatory mechanism of miRNAs involved in the regulation of MAPK cascade genes, their CDS sequences were utilized to predict miRNAs-targeted sites through the online psRNATarget web database (https://www.zhaolab.org/psRNATarget/, accessed on 5 June 2024) with the default parameters [30]. Cytoscape software v 3.9.10 (available at https://cytoscape.org/) was used to generate the schematic diagram depicting the interaction networks between miRNAs and targeted *Saccharum* MAPK cascade genes.

### Proteins interaction network analysis

The String v12.0 online database (available at http://www.string-db.org, accessed on 16 June 2024) was utilized to speculate the proteins-proteins interaction amongst the MAPK cascade genes of *Saccharum* spp. Hybrid R570 and their orthologs in *Zea mays* with default parameters.

### Stress treatments and leaf sample collection

For this study, the sugarcane cultivar Zhongtang3 (ZT3) was sourced from the Institute of Tropical Bioscience and Biotechnology (ITBB), Chinese Academy of Tropical Agricultural Sciences (CATAS), Yazhou Bay Seed Laboratory, located in Sanya, Hainan (56.0411°N, 12.7009°E). Tissue-cultured seedlings of the sugarcane cultivar ZT3 were grown on MS solid medium until they developed 3 to 5 fully unfolded leaves and then transferred to MS solution. After 7–10 days of regeneration, the uniform seedlings were divided into three treatment groups and treated with 20% polyethylene glycol (PEG6000), 250 mmol/L sodium chloride (NaCl) and 50 µM calcium chloride (CaCl_2_). Whole-plant samples were collected for each treatment at 12, 24, 48, and 72 h post-treatment (hpt). Plants grown in MS solution served as controls, with samples collected at 0 hpt. Growth conditions were temperature 30 °C, with a light/dark cycle of 16/8 hours and fixed at 70% relative humidity.

### RNA extraction and qRT-PCR analysis

The Omega (Tiangen Biotech, Beijing Co., Ltd.) RNA extraction kit was used to extract total RNA from sugarcane cultivar ZT3 leaf samples following the procedure established by Ali et al. [2], with minor modifications. Similarly, cDNA synthesis and qPCR programming were performed using the same protocol by Ali et al. [2], with minor adjustments. Primers for six candidate *MAPK* cascade genes (Table S1) were designed with the online PrimerQuest™ Tool (https://sg.idtdna.com/pages/tools/, accessed on 28 June). The *glyceraldehyde-3-phosphate dehydrogenase* (*GAPDH*) gene was used as an internal reference, and the 2^−ΔΔCt^ quantitative method was applied to determine the relative quantitative mRNA profiling.

### Statistical analysis

An analysis of variance (one-way ANOVA) was performed to assess differences in gene expression levels at each time point using IBM SPSS Statistical software. The Post Hoc method with the least significant difference (LSD) test at 0.05 probability level was applied to analyze mean differences at *p* ≤ 0.05. Confidence intervals (95%) were calculated to provide a measure of variability and reliability in the gene expression differences. Data during the experimentation were obtained from the three biological replicates, each containing three technical replicates.

## Results

### Identification of MAPK cascade family genes in polyploid *Saccharum* spp. Hybrid R570 genome

A BLASTp search of the polyploid *Saccharum* R570 hybrid cultivar genome database was performed using 15 ShMAPK, 6 ShMAPKK, and 16 ShMAPKKK protein sequences as query, derived from the monoploid assembly of *Saccharum* spp. Hybrid R570. A total of 89, 24, and 107 genes in the *ScMAPK*, *ScMAPKK*, and *ScMAPKKK* gene families, individually, were identified in the polyploid *Saccharum* spp. R570 genome (Table S2). These genes were renamed according to their chromosomal locations to facilitate systematic identification. Among the 89 predicted ScMAPK proteins, the number of amino acids (aa) varied from 372 (ScMAPK03/05/10) to 746 (ScMAPK52), the predicted isoelectric point (pI) ranged from 5.23 (ScMAPK38) to 9.80 (ScMAPK52), the estimated molecular weight (Mw) ranged from 42.37 kDa (ScMAPK03/05/10) to 83.32 kDa (ScMAPK52), the instability index (II) ranged from 32.24 (ScMAPK31) to 49.95 (ScMAPK52), and the GRAVY values varied between − 0.27 (ScMAPK02/04/06/08/11) and − 0.57 (ScMAPK54). Most genes in the *ScMAPK* family possess a TDY-loop, whereas 18 genes contain a TEY-loop. Sub-cellular localization prediction indicates that the members of the *ScMAPK* family were localized in the cytoplasm, nucleus, chloroplast, or mitochondrial compartment (Table S2). The 24 ScMAPKK proteins were also predicted; the number of amino acids ranged from 347 (ScMAPKK08) to 528 (ScMAPKK18/22), pI ranged from 5.45 (ScMAPKK02) to 6.35 (ScMAPKK08), and putative Mw ranged from 38.77 kDa (ScMAPKK12) to 58.62 kDa (ScMAPKK22), II ranged from 40.93 (ScMAPKK15) to 47.89 (ScMAPKK24) and GRAVY values ranged between − 0.08 (ScMAPKK12) to -0.23 (ScMAPKK22). The *ScMAPKK* family members were predicted to localize in the cytoplasm, mitochondria, or nucleus (Table S2). The 107 ScMAPKKK proteins (subfamilies ScZIK, ScMEKK, and ScRAF) were predicted to consist of 276 (ScRAF04) to 878 (ScMEKK22) amino acids. The pI varied from 4.81 (ScZIK11) to 9.63 (ScRAF03), and Mw varied from 31.01 kDa (ScRAF04) to 98.65 kDa (ScMEKK22). The II ranged from 28.07 (ScRAF42) to 64.28 (ScMEKK01), and GRAVY values ranged between − 0.07 (ScRAF38) to -0.58 (ScZIK05). *ScMAPKKK* family genes were predicted to localize in the chloroplasts, cytoplasm, cytoskeleton, peroxisome, or the nucleus (Table S2).

### Identification of MAP kinase cascades gene clusters in *Saccharum* species

**Fig. 1:**
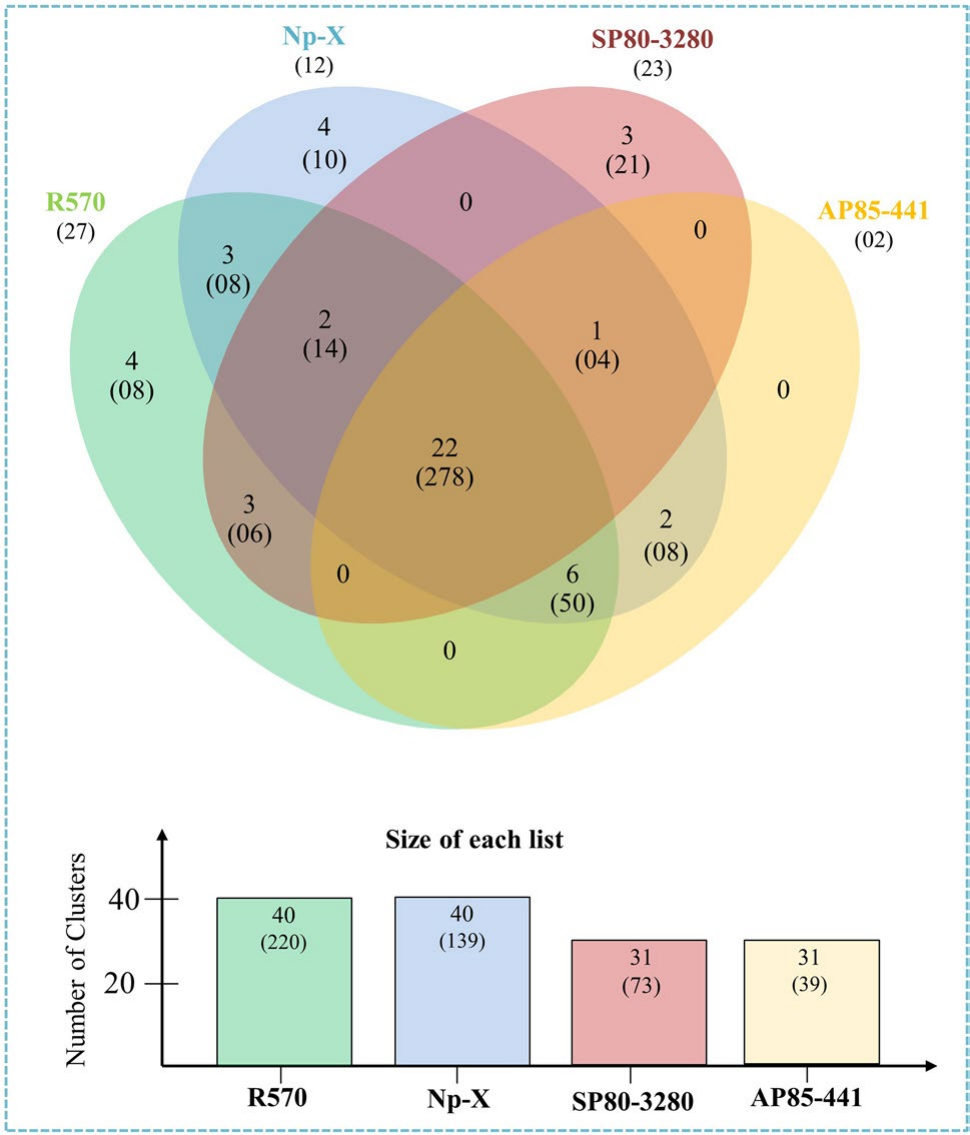
Orthologous clusters of MAPK, MAPKK, MAPKKK family members among *S*. spp. Hybrid R570 (R570), *S. spontaneum* AP85-441 (AP85-441), *S. spontaneum* Np-X (Np-X), and *S*. spp. Hybrid SP80-3280 (SP80-3280). The number in each sector of the diagram indicates the number of homologous clusters and the numbers in parentheses indicate the total number of genes contained within the associated clusters. The numbers in parentheses below the species names indicate the number of species-specific singletons (genes with no homologs)

In this study, a comparative analysis was conducted to detect orthologous clusters and overlapping regions among MAP Kinase cascade genes of *Saccharum* spp. Hybrid R570, the two *S. spontaneum* clones (AP85-441and Np-X) and *Saccharum* spp. Hybrid SP80-3280 (Fig. 1). Overall, 40 (220 proteins), 40 (139 proteins), 31 (73 proteins), and 31 (39 proteins) orthologous MAP kinase cascade clusters were observed in R570, AP85-441, Np-X and SP80-3280, respectively. Among them, a total of 22 clusters (278 proteins) of MAP Kinase cascade genes were displayed in all four genomes. In contrast, six clusters (50 proteins) were common in two *S. spontaneum* genomes (AP85-441 and Np-X) and the *Saccharum* spp. Hybrid R570 genome. Surprisingly, four clusters (18 proteins) and three clusters (21 proteins) were identified exclusively in R570, Np-X, and SP80-3280 genomes, respectively. Interestingly, 64 singletons were also detected in four *saccharum* genomes. Of these, 27 in R570, 23 in SP80-3280, 12 in Np-X, and two singletons were found in the AP85-441 genome, respectively (Fig. 1).

### Comparative phylogenetic analysis of MAP kinase cascade genes in different plant species

Comprehensive phylogenetic analyses of MAPK, MAPKK, and MAPKKK gene families from *Saccharum* spp. Hybrid R570 (Sc), *Z. mays* (Zm), *A. thaliana* (At), *J. curcas* (Jc), and *G. raimondii* (Gr) are presented in Figure S1. Comparative phylogenetic analysis highlighted that all 127 MAPK genes could be categorized into five distinct groups (I-V). Group I was the largest clade, comprising 51 MAPKs, followed by Group II with 33 genes, Group V with 19 genes, and Group IV with 17 genes. The smallest clade was Group III, containing only six genes (Figure S1A). In addition, the 43 MAPKKs were classified into four different groups (I to IV), with 14 members in Group I, 10 in Group III and IV, and 9 in Group II (Figure S1B). All 381 MAPKKK members from numerous plants were grouped into three main subfamilies: ZIK-like, MEKK-like and RAF-like, based on their specific conserved domains (Figure S1C). The MAPKKK RAF-like subfamily included 44 genes from *Sc*, 34 genes from *Zm*, 43 genes from *At*, 36 genes from *Jc*, and 46 genes from *Gr*. The MAPKKK MEKK-like subfamily consisted of 42 genes from *Sc*, 19 genes from *Zm*, 21 genes from *At*, 12 genes from *Jc*, and 24 genes from *Gr*. Similarly, the MAPKKK ZIK-like subfamily comprised 21 genes from *Sc*, two from *Zm*, nine from *Jc*, 11 from *At*, and 14 from *Gr* (Figure S1C).

### Phylogenetic, conserved domain, gene structure, and motif prediction of *ScMAPKs*

**Fig. 2:**
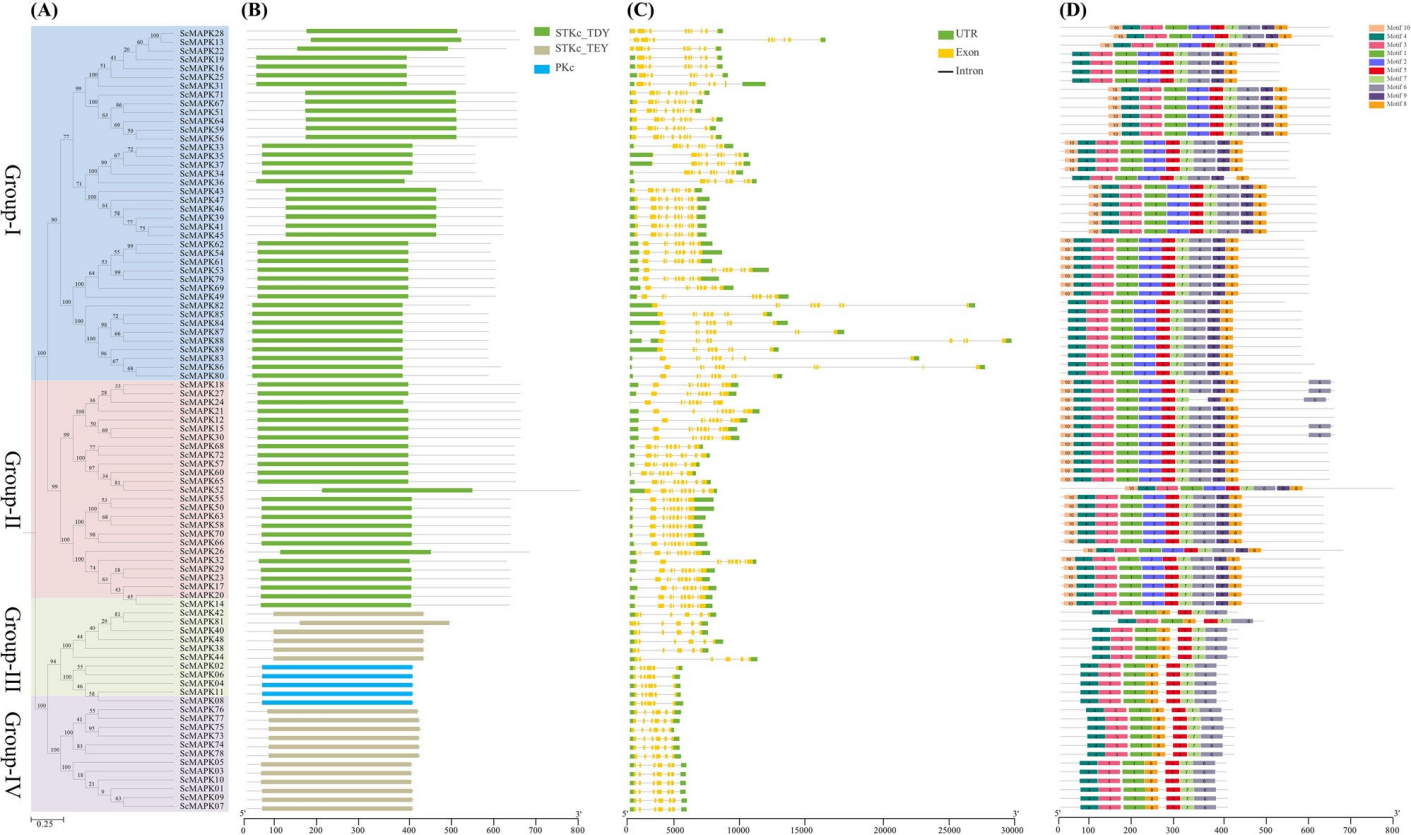
Phylogenetic relationships (**A**), Conserved domains (**B**), Exon-intron structures (**C**), and Conserved motifs (**D**) of ScMAPK family genes from *Saccharum*. spp. Hybrid R570. (**A**) Four phylogenetic groups (I, II, III and IV) with different colored backgrounds were observed. (**B**) The functional domains (STKc_TDY, STKc_TEY and PKc) were obtained from model gene annotation and results of the NCBI CDD-batch search. (**C**) The introns, exons and UTRs in each gene are represented by gray lines, yellow and green boxes, respectively. (**D**) Conserved motifs (1-10) were identified using MEME and denoted by different colors. Protein length is estimated using the scale (amino acid) in (**B**) and (**D**) panels, while gene length is estimated using the scale (bp) at the bottom in (**C**) panel

A phylogenetic tree was constructed with 89 *ScMAPK* gene members identified from *Saccharum* spp. Hybrid R570. All *ScMAPK* family genes were divided into four Groups (I: IV), i.e., 40 genes in Group I, 25 in Group II, 11 in Group III, and 13 in Group IV (Fig. 2A). Among the *ScMAPK* family members, 66 and 18 members contained a Serine/Threonine kinase, Plant-TDY/ or -TEY catalytic domain (STKc_TDY/or _TEY; NCBI-CDD accession number: CD07859/ or CD07834), and five genes (*ScMAPK02*/*04*/*06*/*08*/*11*) harbored Protein kinase C domain (PKc; NCBI-CDD accession number: CD07834) (Fig. 2B). We analyzed the gene structure to further explore the conservation of *ScMAPK* family genes (Fig. 2C). All 89 MAP kinase cascade genes had both 5ˊ- and 3ˊ-UTRs regions. Gene structure investigation also displayed that *ScMAPK* family genes in diverse phylogenetic clades had amazingly changed exon-intron structures. The number of exons ranged from 6 (Group III and IV) to 11 (Group I), while the introns count varied between 5 (Group III and IV) and 9 (Group I and II). Notably, four genes (*ScMAPK82*/*83*/*86*/*88*) from Group I possessed the longest introns, while the longest exons were detected in Group II. Interestingly, our analysis revealed that the position and number of exons and introns were largely consistent among genes within the same group or subgroup (Fig. 2C). Using the online MEME web tool, we also analyzed conserved motifs in *ScMAPK* cascade genes (Fig. 2D). A total of ten motifs were detected, of which seven (motif1/3/4/5/6/7/8) were predicted to almost all *ScMAPK* genes. In Group I and II, most *ScMAPK* genes contained motifs 9 to 10. Interestingly, Group III and IV contained only seven common motifs, lacking motifs 2, 9, and 10. However, motif 10 was explicitly observed in almost all member genes of Group II, while motif 2 was predominantly found in Group I, and II genes only (Fig. 2D).

### Phylogenetic, conserved domain, gene structure, and motif analysis of *ScMAPKKs*

**Fig. 3:**
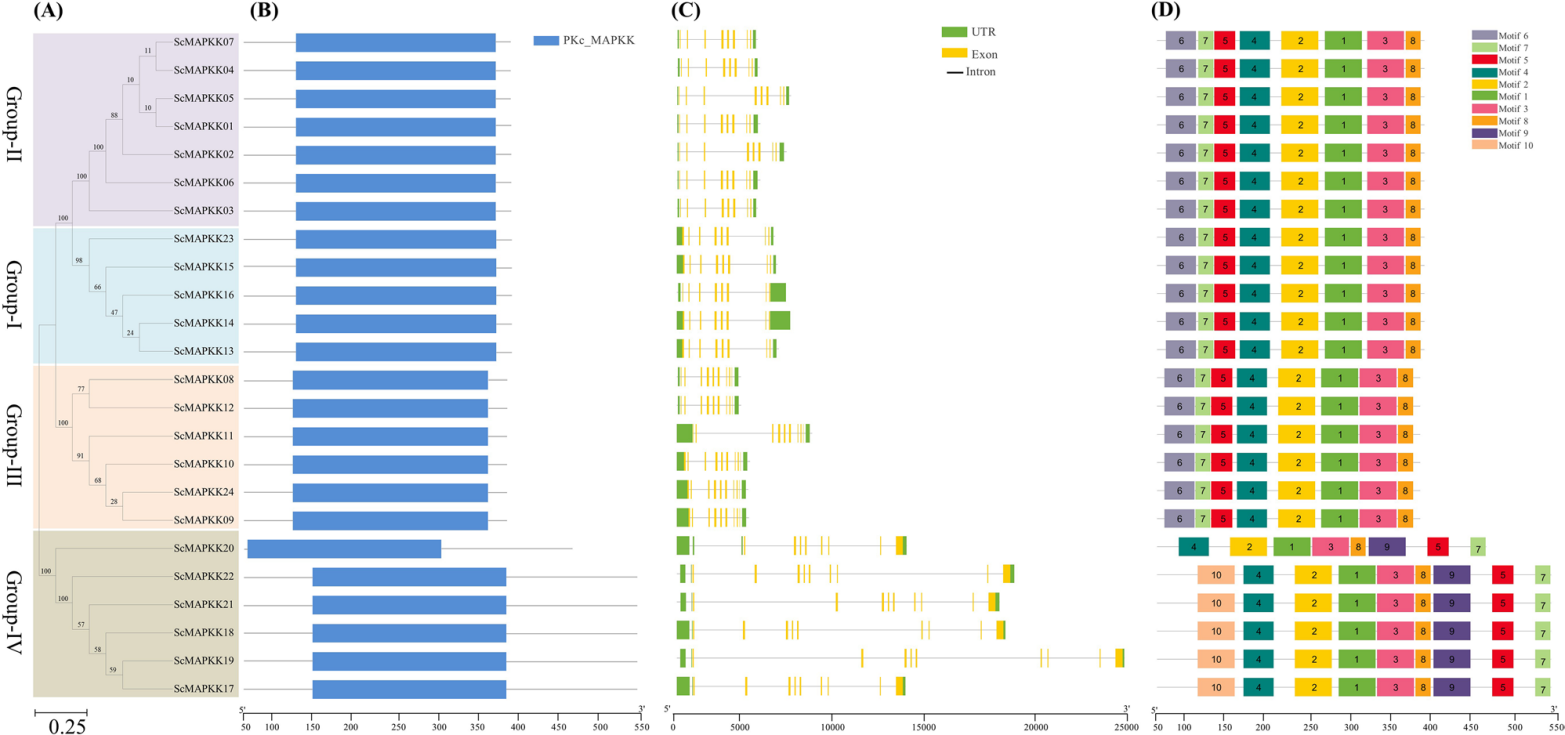
Phylogenetic relationships (**A**), Conserved domains (**B**), Exon-intron structures (**C**), and Conserved motifs (**D**) of ScMAPKK family genes from *Saccharum* spp. Hybrid R570. (**A**) Four phylogenetic groups (I, II, III and IV) with different colored backgrounds were observed. (**B**) The functional domain (PKc-MAPKK) was obtained from model gene annotation and results of the NCBI CDD-batch search. (**C**) The introns, exons and UTRs in each gene are represented by gray lines, yellow and green boxes, respectively. (**D**) Conserved motifs (1-10) were identified using MEME and denoted by different colors. Protein length is estimated using the scale (amino acid) in (**B**) and (**D**) panels, while gene length is estimated using the scale (bp) at the bottom in (**C**) panel

Phylogenetic analysis categorized the 24 *ScMAPKK* genes into four (I: IV) significant groups (Fig. 3A). Group I comprise seven *ScMAPKK* members (*ScMAPKK01*/*02*/*03*/*04*/*05*/*06*/*07*) genes, while Group II contains five members (*ScMAPKK13*/*14*/*15*/*16*/*23*). Group III includes six members (*ScMAPKK08*/*09*/*10*/*11*/*12*/*24*), and Group IV also comprises six members (*ScMAPKK17*/*18*/*19*/*20*/*21*/*22*) (Fig. 3A). The prediction of conserved domains (CD) highlighted that almost all ScMAPKK proteins possess the dual-specificity Protein Kinase C, MAP kinase kinase (PKc_MAPKK; NCBI-CDD accession number: CD06605) superfamily domain (Fig. 3B). The gene structure study discovered that most of the *ScMAPKK* genes contained between 8 and 9 exons and 10 to 12 introns (Fig. 3C). Group I members typically have 8 exons and 10 introns, indicating a relatively simpler structure. In contrast, Group II, Group III, and Group IV members display more complex gene structures, each possessing 9 exons and 12 introns. Notably, *ScMAPKK08* and *ScMAPKK12* show distinct structures with 9 exons and 12 introns, marking significant differences from other groups. This implies that members within the same group generally share similar genetic architectures. Moreover, most genes within Group III exhibit similar exon-intron structures except for *ScMAPKK11*, which shows some variation. In addition, all *ScMAPKK* members of Group IV have more complex gene structures, with longer introns and exons lengths than other groups. In particular, *ScMAPKK19* contains two of the longest intron sequences, underscoring its structural complexity (Fig. 3C). To further investigate the conserved motifs within *ScMAPKK* family members, the online MEME web server was used to predict their distribution. A total of 10 motifs were projected in the identified *ScMAPKK* family member genes (Fig. 3D). Group I, II, III, and IV members shared a common set of seven (motif 1/2/3/4/5/7/8) in the same order, indicating a core structure conserved across the family. However, motifs 9 and 10 were absent in Group I, II, and III but were specifically present in Group IV *ScMAPKK* members. Interestingly, motif 6 was detected only in Group I, II, and III and absent from Group IV. Overall, members within the same phylogenetic clade tend to exhibit similar motif distributions (Fig. 3D).

### Phylogenetic, conserved domain, gene structure, and motif analysis of *ScMAPKKKs*

**Fig. 4:**
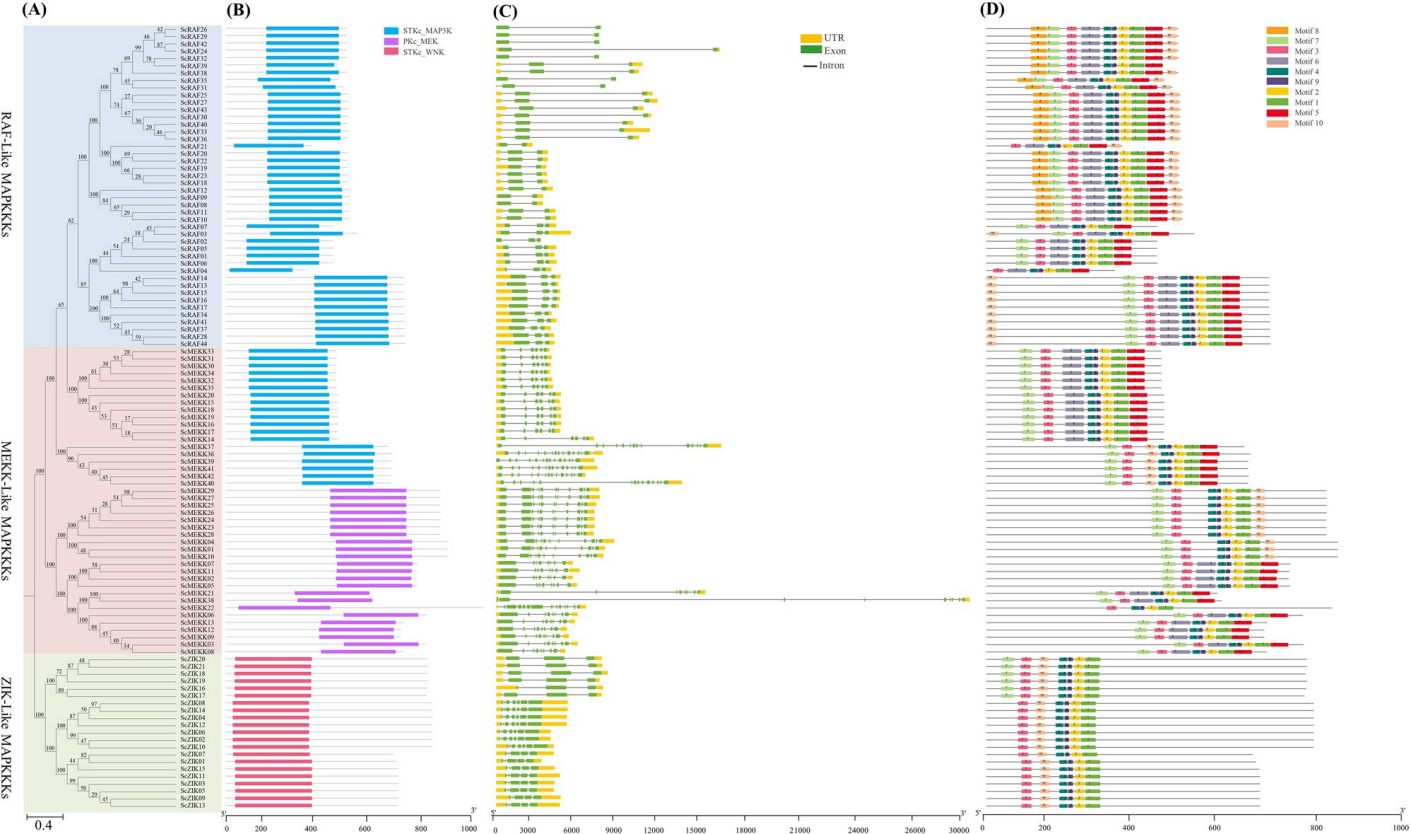
Phylogenetic relationships (**A**), Conserved domains (**B**), Exon-intron structures (**C**), and Conserved motifs (**D**) of ScMAPKKK family genes from *Saccharum* spp. Hybrid R570. (**A**) Different color codes represent three groups (Raf, MEKK, and ZIK). (**B**) The functional domains (STKc_MAP3K, PKc_MEK, and STKc_WNK) were obtained from model gene annotation and results of the NCBI CDD-batch search. (**C**) The introns, exons and UTRs in each gene are represented by gray lines, yellow and green boxes, respectively. (**D**) Conserved motifs (1-10) were identified using MEME and denoted by different colors. Protein length is estimated using the scale (amino acid) in (**B**) and (**D**) panels, while gene length is estimated using the scale (bp) at the bottom in (**C**) panel

We analyzed the evolutionary relationship within the *ScMAPKKK* family by constructing an evolutionary phylogenetic tree of 107 *ScMAPKKK* family member genes (Fig. 4A). This analysis revealed that the *ScMAPKKK* gene family in *Saccharum* spp. Hybrid R570 can be separated into three main subfamilies: ZIK-like, MEKK-like and RAF-like MAPKKKs, based on the presence of family-specific conserved domains (CD). Specifically, the RAF-like subfamily contains 44 members, the MEKK-like subfamily includes 42 members, and the ZIK-like subfamily consists of 21 members (Fig. 4A). Conserved domain analysis of *ScMAPKKKs* shows that all the members associated with RAF like subfamily and 19 members from the MEKK-like subfamily contain a ser/thr kinase, MAP Kinase Kinase Kinase (STKc_MAP3K; NCBI-CDD accession number: CD14172). In contrast, 23 genes of MEKK-like MAPKKKs genes possess a catalytic domain of the dual-specificity P kinase, MAP/extracellular signal-regulated kinase (ERK) kinase (PKc_MEK; NCBI-CDD accession number: CD06615). Additionally, all member of the ZIK-like subfamily contains a ser/thr kinase domain with no lysine (WNK) kinase (STKc_WNK; NCBI-CDD accession number: CD13983) (Fig. 4B). To examine the structural features of *ScMAPKKKs*, we analyzed intron-exon organization using GSDS and TBtools-II software. The intron/exon structures of the *ScMAPKKK* gene family members illustrated in Fig. 4C reveal that these genes contain between 2 exons (in RAF-like subfamily members) and 14 exons (*ScMEKK36*/*37*/*39*/*40*/*41*/*42*), with intron numbers ranging from 1 (*ScRAF08*/*09*/*24*/*26*/*29*/*32*/*35*/*42*) to 15 introns. Most genes in the MEKK-like subfamily have more than 10 introns, while the intron counts in the ZIK-like subfamily range from 3 to 6. The MEKK-like and ZIK-like subfamilies exhibit more complex structures than the RAF-like family, mainly due to greater intron and exon number variation. Generally, *ScMAPKKKs* within the same phylogenetic cluster tend to share similar exon-intron structures, signifying the connection between evolutionary relationships and gene structure. Notably, *ScMEKK38* in the MEKK subfamily features the longest introns, while genes in the RAF-like subfamily had the longest exons (Fig. 4C). A motif hunt was carried out by utilizing the online web server to investigate the evolutionary characteristics of the *ScMAPKKK* family member sequences (Fig. 4D). Ten conserved motifs were predicted in RAF-like, MEKK-like, and ZIK-like subfamilies. Nearly all the *ScMAPKKK* genes contained the core protein kinase domain with motifs 1, 2, and 3. Motif 4 and motif 9 were conserved across all the proteins, while motifs 5 and 6 were unique to the MEKK-like and RAF-like subfamilies. Conversely, the ZIK-like subfamily predominantly contains motifs 1, 2, 3, 9, and 10, but lacks motifs 5, 6, 7, and 8, except for six members (*ScZIK16*/*17*/*18*/*19*/*20*/*21*) that include motif 7. Notably, motif 8 was only specific to the RAF-like subfamily. Interestingly, one MEKK-like subfamily member, *ScMEKK22*, possesses only 5 motifs (motifs 1, 2, 3,4, and 9) (Fig. 4D).

### Multiple sequence alignment and *cis*-regulatory elements analysis

Multiple sequence alignment examination highlighted that all 89 identified ScMAPKs contained 10 conserved subdomains (I to X), with essential STKc and PKc motifs, a C-terminal and activation loop (T-loop) motifs (Figure S2.A). For instance, threonine (T) and tyrosine (Y) residues in the TEY, MEY, or TDY motifs within the T-loop regions were present between subdomains V and VIII. The C-terminal motif is a key site for phosphorylation during the activation of MAP kinase. Furthermore, all ScMAPKs exhibit a conserved -T/M(E/D)Y- motif: specifically, 16 ScMAPKs contain the TEY motif, six show the MEY motif, and 67 featured the TDY motif (Figure S2.A). The 24 ScMAPKKs shared 5 conserved motifs, with the active site {[D-[L/I/V/M]-K]} motif and the T-loop (S/T-xxxxx-S/T) as well as the VGT-2x-YM-x-PER, D/I-3x-G/L, and FPY sub-domain motifs, which plays important roles in signal transduction (Figure S2.B). Among the 107 ScMAPKKKs, the RAF-like subfamily shared the conserved motif GT-2x-(W/Y)-MAPE, which confirmed their association with the RAF subfamily. The MEKK-like subfamily harbored a distinct G-(S/T)-P-x-(Y/W)-MAPEV motif, which strongly supported association within the RAF-like subfamily. The ZIK-like subfamily, associated with ZIK-like kinases [or WNK kinases, with no lysine (K)], exhibits the conserved GTPEFMAPE-(M/V) signature motif (Figure S2.C).

The *cis*-regulatory elements analysis of the 2.0 kb upstream promoter regions of MAPK cascade genes was performed to elucidate the roles of *ScMAPK*, *ScMAPKK*, and *ScMAPKK* genes in biotic and abiotic stress responses (Figure S3). A total of 20,089 *cis*-elements, categorized into 22 subtypes associated with abiotic and biotic stressor responses, were recognized within the 2.0 kb promoter regions of 220 MAPK cascade genes in *Saccharum* spp. Hybrid R570 (Figure S3.1). These elements were further categorized into four major groups based on their function: light response-related, hormone-related, stress-related, and growth and development-related elements. Of these, 14.0% (2723/20089) were light response-related, 19.0% (3841/20089) were hormone-related, and 62.0% (12492/20089) elements were associated with stress-related. Only 5.0% (1033/20089) elements were linked to growth and development (Figure S3.1). Interestingly, the promoter regions of *ScMAPK07*/66, *ScMAPKK13*, *ScZIK18*, and *ScRAF10*/*43* were found to contain several *cis*-regulatory elements associated with stress responses. For example, *ScMAPK07*/66 and *ScMAPKK13* had numerous *cis*-elements, including ARE, TATA-box, MBS, STRE, as-1, Myb-binding site, MYB, GC-motif and W-box. In addition, *ScZIK18* and *ScRAF10*/*43* contained LTR, MBS, DRE core, MYB, MYB recognition site, as-1, MYC, STRE, TATA-box, and GC-motifs (Figure S3.1).

Furthermore, among MAPK cascade genes, 219 genes confined the defense and stress-related elements, whereas 218 genes contained light response-related and hormones response-related. In comparison, only 216 genes hold growth and development-related elements (Figure S3.2A). The four major groups were further divided into sub-groups based on the occurrence of different elements. The hormone response-related category further includes ABRE (abscisic acid-responsive), TCA-element (response to morphogenesis), CGTCA-motif (involved in response to methyl jasmonate), TGA-element (response to auxin), and TGACG-motif (response to MeJA). The highest number of different *cis*-elements from the hormone response-related category was ABRE (27%) followed by (25%) of CGTCA-motif, while the ERE and TATC-box consisted of only 2% and 1% elements, respectively (Figure S3.2B). Light response-related category comprised of the G-box (involved in gene expression), Sp1 (response to light), AE-Box (light-responsive element), Box-4 (response to light conditions), and GATA-motif (involved in expression of genes to light) elements. The highest light-response related *cis*-elements were G-Box (42%) followed by Sp1 (21%), while the Box-4 consisted of only 9% elements (Figure S3.2C). The growth and development-related category includes CAT-box (response to cold or dehydration conditions), GT1-motif (light-responsive), Circadian (involved in photosynthetic activity), ARE (response to abscisic acid), GCN4-motif (involved in endosperm-specific), and RY-element (controlling seed maturation). The highest number of *cis*-elements from the growth and development response-related category was ARE (45%), followed by CAT-box (33%), while the HD-Zip1 and motif1 consisted of only 1% *cis*-elements (Figure S3.2D). The stresses-related category included ARE (anaerobic induction), MYB-binding site (drought or light-responsive), DRE core (dehydration-responsive), MYC (response to drought), W-box (response to wounding), WUN-motif (response to wound), as-1 (response to oxidative stress), MYB (stress-related), STRE (stress-related), and TATA-box (stress-related). The highest defense-related *cis*-elements consisted of TATA-Box (39%), followed by 13% of MYB, while the STRE and MYC consisted of 10% and 9% *cis*-elements, respectively (Figure S3.2E).

### Gene location, duplication events and syntenic relationship of MAPK cascade genes

The distribution of *ScMPAK*, *ScMAPKK*, and *ScMAPKKK* genes in the polyploid genome of *Saccharum* spp. Hybrid R570 is presented in supplementary Figure S4. The majority of MAPK cascade genes are located on the proximate or distal ends of the chromosomes. All 220 MAPK cascade genes were unevenly distributed across nine main chromosomes (63 sub-chromosomes) and four scaffolds in the R570 genome. Specifically, seven genes (4 *ScMAPKs*, 1 *ScMAPKK*, 1 *ScRAF*, and 1 *ScMEKK*) were located on chromosome Chr6A/F/_9A, while six MAPK cascade genes were present on 10 chromosomes (Chr2A-G, Chr6C, and Chr8D/_10A) each. Additionally, a single MAPK cascade gene was found on 13 individual sub-chromosomes (Chr3A/E, Chr4F, Chr6B, Chr7A-E/os1, and Chr10E/F/os1/2) and one scaffold (scaffolod_92). Interestingly, chromosome 2 contained the highest number of MAPK cascade genes, with a total of 43 (21 *ScMAPKs*, 07 *ScMAPKKs*, 08 *ScMEKKs*, and 07 *ScRAFs*), followed by chromosome 6, which harbored 39 genes (24 *ScMAPKs*, 03 *ScMAPKKs*, 05 *ScMEKKs*, and 04 *ScRAFs*). However, no MAPK cascade genes were detected in the remaining 23 sub-chromosomes and 47 scaffolds of *Saccharum* spp. R570 genome (Figure S4).

**Fig. 5:**
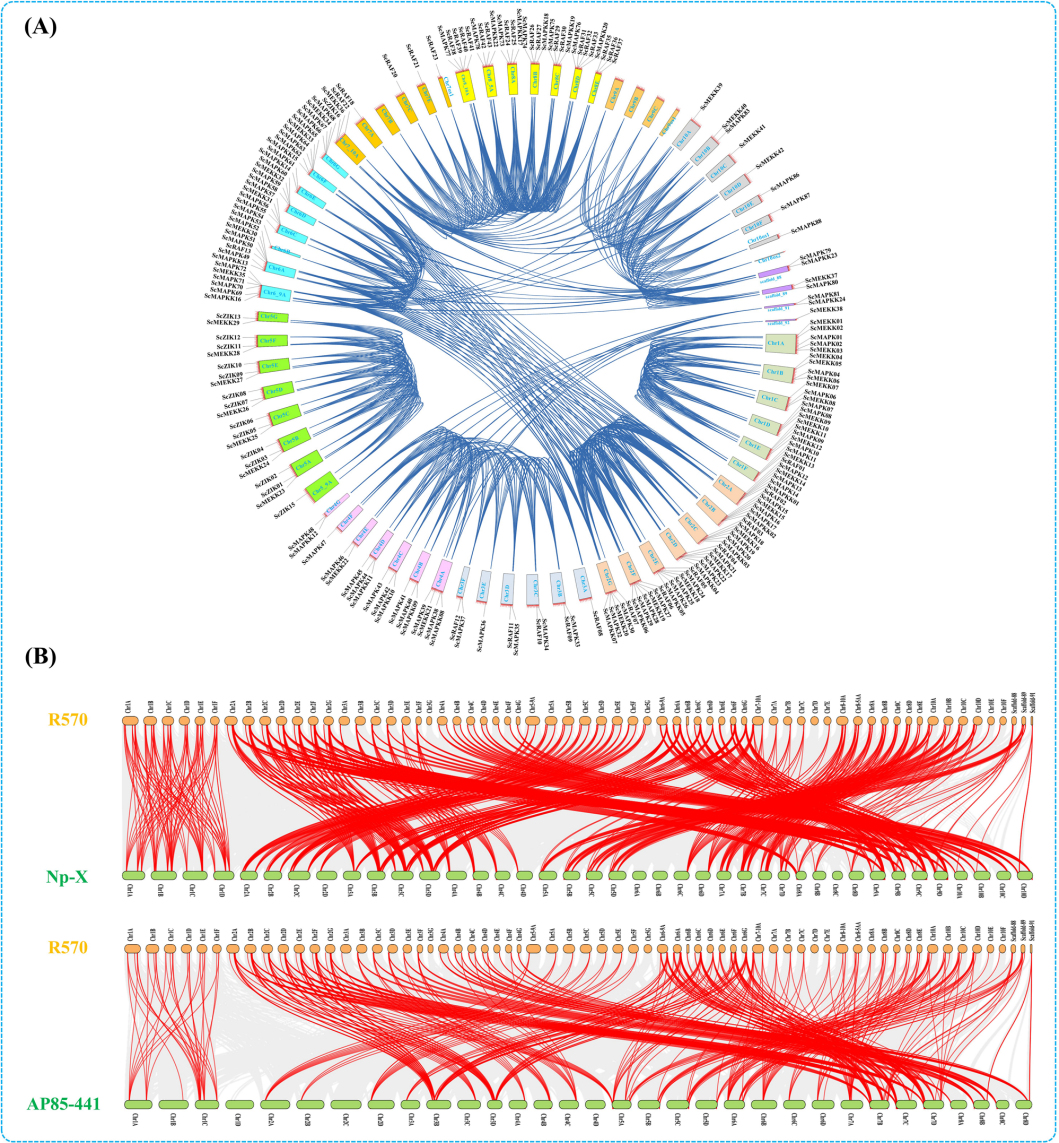
Gene duplication and Synteny analysis of MAP kinase families gene. (**A**) Circos illustration of duplicated *ScMAPK*, *ScMAPKK*, and *ScMAPKKK* genes on chromosomes of *Sarcharum* spp. Hybrid R570. The dark blue lines show gene paralogs. (**B**) Multicollinearity analysis of MAP kinase families gene in three genomes (R570 *vs* Np-X and R570 *vs* AP85-441). The chromosomes are arranged in a colored arc. Red lines represent the syntenic relationship of MAP kinase genes, and gray lines in the background represent all orthologous genes in these genomes

An analysis of gene duplication and collinearity in all MAPK cascade genes showed that out of 220 pairings (76.81%), allelic and non-allelic segmental duplications were likely implicated in 169 pairs of genes (Fig. 5A). Gene duplication was observed in all 21 pairs of non-allelic duplicated genes on Chr5_9A-Chr5G, while 22 pairs of non-allelic duplicated genes on Chr1A-Chr1F. In addition, 32 and 27 allelic duplication events were found on several chromosomes and scaffolds, respectively. At the same time, 63 MAPK cascade genes (14 *ScMAPKs*, 08 *ScMAPKKs*, 27 *ScMEKKs*, 07 *ScRAFs*, and 07 *ScZIKs*) were duplicated on the unidentified regions of chromosome and scaffolds. Notably, chromosomes Chr6A-G and Chr2A-G were hot zones for duplication, as proved by 70 segmentally (allelic and non-allelic) duplicated pairs. A single duplicated gene pair was found on chromosomes Chr3A/E, Chr4F, Chr5_9A, Chr6B, Chr7A/C/F/os1, Chr10A/C-F/os1, and one scaffold_92. However, no duplicated gene pairs were detected on chromosomes Chr7B, Chr9A-C/os1, and Chr10os2. No tandem duplication existed on *Saccharum* spp. Hybrid R570 genome (Fig. 5A).

Comparative synteny analysis among *Saccharum* spp. Hybrid R570 (R570) and the two *S. spontaneum* clones (Np-X and AP85-441) showed that R570 and Np-X had notable syntenic relations with each other and AP85-441 (Fig. 5B). A total of 212 MAPK cascade family members from R570 shared syntenic relationships in the Np-X genome, and 180 members had syntenic relationships in the AP85-441 genome. Notably, no synthetic relationship was detected for *ScMEKK21*/*22*/*32*/*38* and *ScRAF27*/*32*/*40*/*43* between R570 vs. Np-X, and for *ScMAPK73*/*77*/*77*, *ScMAPKK17*/*18*/*19*/*20*/*21*/*22*, *ScMEKK1*/*4*/*10*/*21*–*29*/*32*/*38*, *ScRAF25*/*27*/*30*/*32*/*33*/*36*/*39*/*40*/*43* and *ScZIK1*/*3*/*5*/*7*/*9*/*11*/*13*/*15* between R570 vs. AP85-441 (Fig. 5B and Table S3).

### MiRNA prediction and protein-protein interactions

**Fig. 6:**
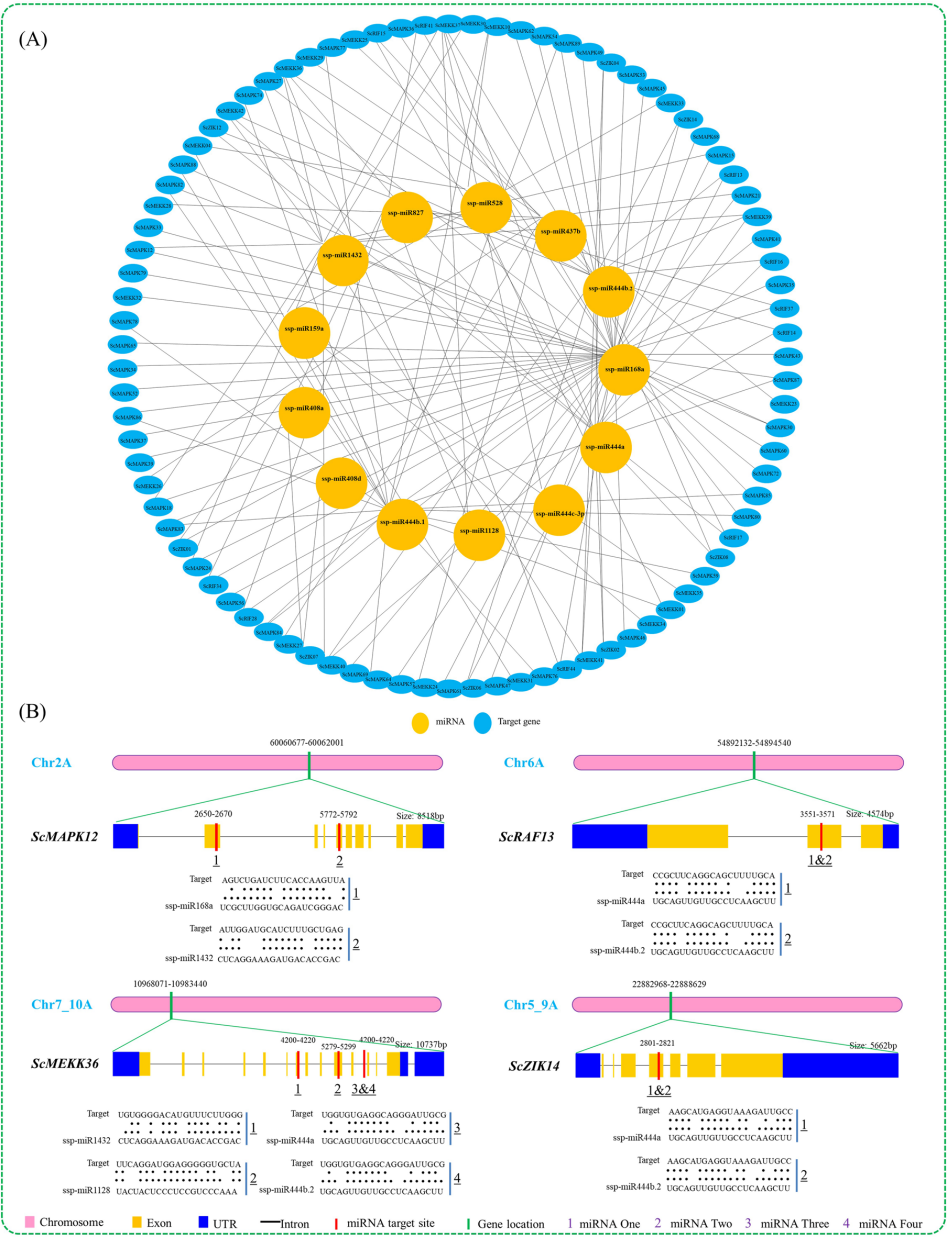
Network map of predicted miRNAs targeting MAP kinase families gene (**A**) and schematic diagram of miRNAs targeting sites in some MAP kinase (**B**). (**A**) Blue and orange boxes stand for miRNAs and their targeted genes of MAP kinase families gene. (**B**) The thick green bar indicates the MAPK genes location at the specific chromosome. The thick red bar indicates the location of the miRNA targeted at the gene sequence. The RNA sequence of each complementary site from 5’ to 3’ and the predicted miRNA sequence from 3’ to 5’ are shown with green lines below the gene sequences. All miRNAs predicted to target MAP kinase family genes are shown in Supplementary Table S4. Ssp; *Saccharum* spp. Hybrid

To better understand the roles of miRNAs in managing the post-transcriptional regulation of MAPK cascade genes, the CDS sequences of 89 *ScMAPKs*, 24 *ScMAPKKs*, and 107 *ScMAPKKs* were examined in the online psRNATarget web server against the 20 published miRNAs of *Saccharum* spp. Hybrid (ssp.). A total of 87 genes (47 *ScMAPKs*, 22 *ScMEKKs*, 10 *ScRAFs* and 8 *ScZIKs*) were targeted by 13 unique miRNAs that fit into nine different families (Fig. 6A and Table S4). One miRNA, ‘ssp-miR168a’ targeted 45 genes (40 *ScMAPKs* and five *ScRAFs*). The three miRNAs “ssp-miR444a”, “ssp-miR444b.1”, and “ssp-miR444b.2” targeted 17 (six *ScMEKKs*, five *ScRAFs* and six *ScZIKs*), 19 (16 *ScMAPKs* and three *ScMEKKs*), 17 (six *ScMEKKs*, five *ScRAFs* and six *ScZIKs*) genes from *Saccharum* spp. R570 respectively. The miRNA ‘ssp-miR528’ targeted three *ScMEKK* genes. The miRNA ‘ssp-miR827’ targeted five *ScRAF* genes. The miRNA ‘ssp-miR1128’ targeted *ScMEKK36* and *ScMEKK37* genes from the ScMAPKKK family. On the other hand, *ScMEKK37* was targeted by five miRNAs, while four miRNAs targeted *ScMEKK36*. In addition, *ScMEKK39*/*40*/*41*/*42* were targeted by only three miRNAs each. Interestingly, 44 genes, 31 *ScMAPKs*, and 13 *ScMEKKs* were targeted by a single miRNA each (Fig. 6A and Table S4). The representative targeted sites of miRNA in *ScMAPK12*, *ScMEKK36*, *ScRAF13*, and *ScZIK14* are illustrated in Fig. 6B.

**Fig. 7:**
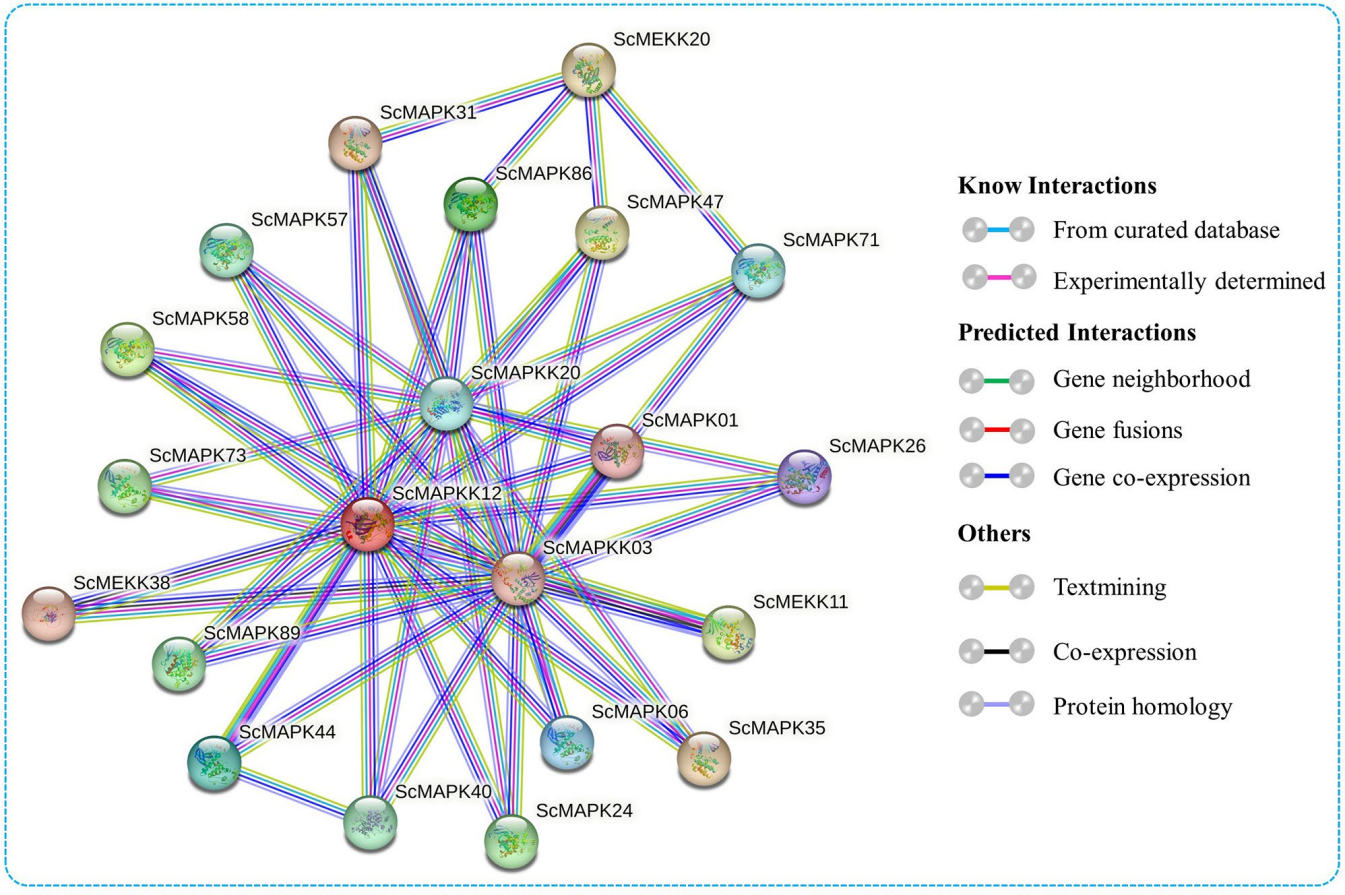
Predicted protein-protein interactions of ScMAPK, ScMAPKK and ScMAPKKK proteins according to their homologous pair with *Zea mays* in STRING database. In the network, only the pairs >50% sequence identity between *Saccharum* spp. Hybrid R570 and *Z. mays* MAPK kinase family proteins and with an interaction score > 0.8 are shown. Line and node colors indicate the different kinds and degrees of interactions, respectively. The details of the protein-protein interaction network are available in Table S5

To investigate the possible biological functions of ScMAPK, ScMAPKK, and ScMAPKKK proteins, we utilized the STRING online web tool to predict their protein-protein interaction (PPI) network (Fig. 7 and Table S5). MAPK cascade proteins with higher homologous similarity (%) and bit scores to *Zea mays* proteins were selected for network analysis. The PPI network analysis found that 21 protein pairs had interactions between ScMAPK, ScMAPKK, and ScMAPKKK family proteins. Among them, ScMAPKK12 (homologous of maize Mek1) and ScMAPKK03/20 (homologous of maize Mek1and A0A1D6MU28) were positioned in the middle of the proteins network and showed interaction with 16 and 15 proteins in the network, respectively. Furthermore, ScMEKK20 (homologous of maize A0A1D6NHP1) interacted with four (ScMAPK31/71/86/47) MAPK cascade proteins. Interestingly, ScMAPK31/40/44/47/86 proteins (homologous of maize K7VK23_MAIZE, MPK7, Mpk2, A0A1D6GVT0, and A0A1D6F0V1) interacted with four MAPK proteins each in the network (Fig. 7). Additionally, the interaction network analysis indicated that MAPK cascade members are involved in various biological processes, including plant innate immune responses, responses to diverse environmental stressors, defense mechanisms, regulation of cellular processes and response to abiotic stressors (Fig. 7 and Table S5).

### Transcript expression of MAPK cascade genes under abiotic stressors

**Fig. 8:**
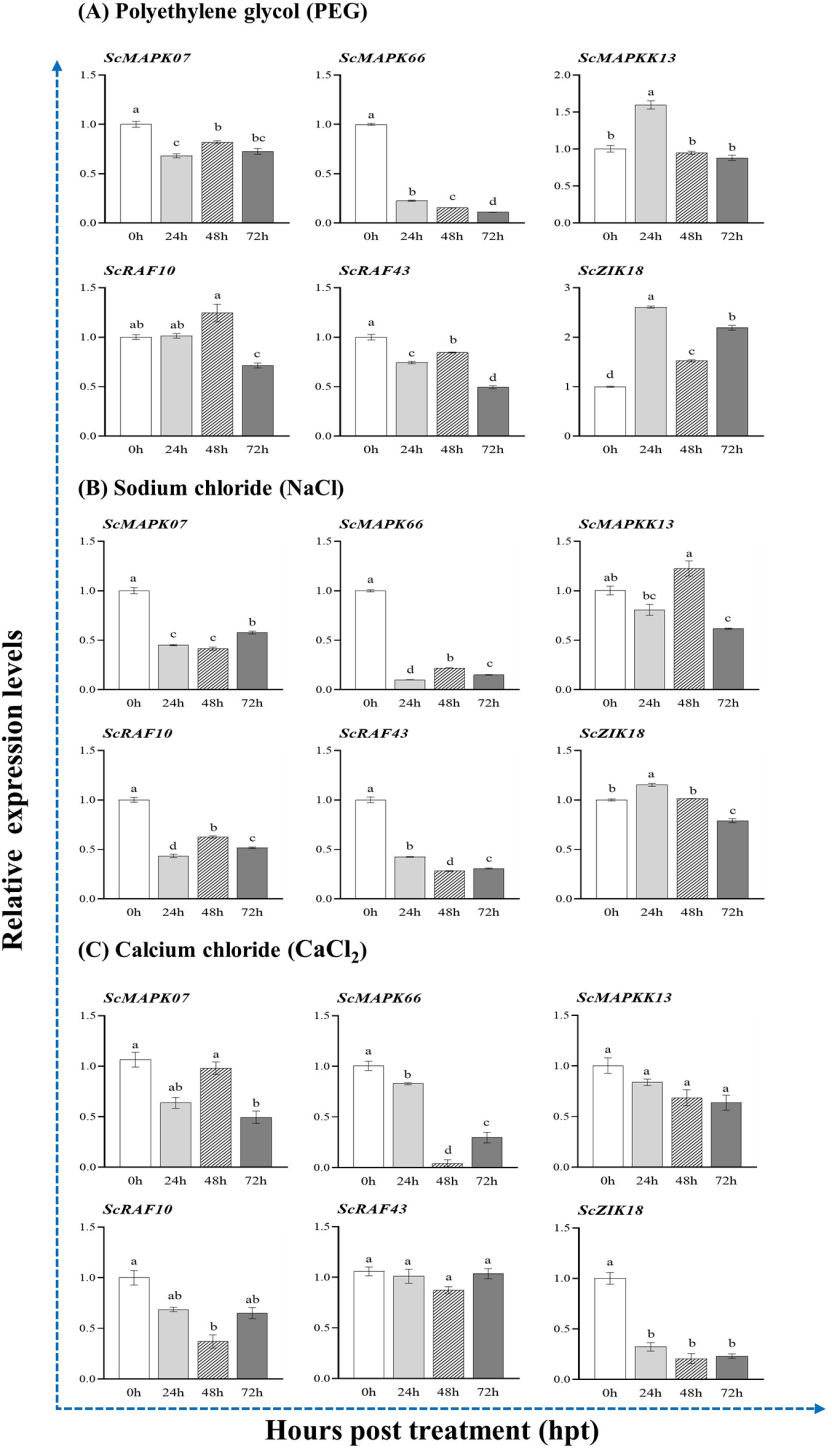
Expression analysis of MAPK cascade genes at different treatment time points in response to abiotic stresses in sugarcane cultivar ZhongTang3 (ZT3). (**A**) Drought (PEG6000) stress; (**B**) Salinity (NaCl) stress, and (**C**) Calcium chloride (CaCl_2_) stress. Vertical bars represent the mean ± standard error (three replicates). Different letters (a, b, c, d) on the bar represent significant differences among treatments following LSD test (*p* < 0.05). The 0 h indicates control treatment (mean value =1). Significance differences and confidence intervals values are provided in Table S6

Transcript profiles of six genes (*ScMAPK07*/*66*, *ScMAPKK13*, *ScRAF10*/*43*, and *ScZIK18*) were explored in sugarcane cultivar Zongtang3 (ZT3) under PEG, NaCl and CaCl_2_ stress treatments based on qRT-PCR assays (Fig. 8). Under drought (PEG) treatment, the transcript levels of three genes (*ScMAPK07*, *ScMAPK66* and *ScRAF43*) were reduced, but the transcript patterns of two genes, *ScMAPKK13* and *ScRAF10*, were significantly elevated by 1.5-fold and 1.2-fold, particularly at 24 and 48 hpt, while remaining unchanged or down-regulated across other treatments time points. The expression of *ScZIK18* was remarkably up-regulated by 1.5- to 2.6-fold in the Zhongtang3 cultivar across all time points after PEG treatment, as compared with the mock-treatment 0 hpt (Fig. 8A). Our findings suggest that *ScZIK18* is a defense-related gene in sugarcane against PEG stress. Under salinity (NaCl) treatment, four genes, *ScMAPK07*/*66*, *ScRAF10*, and *ScRAF43*, were significantly down-regulated across all time points, and *ScZIK18* was not significantly changed as compared with the control treatment at 0 hpt in Zhongtang3. The two genes *ScZIK18* and *ScMAPKK13* were up-regulated with an increase of 1.1- and 1.3-fold at 24 and 48 hpt under NaCl treatment (Fig. 8B). In addition, under the calcium (CaCl_2_) treatment, five genes (*ScMAPK07*/*66*, *ScMAPKK13*, *ScRAF10*, *ScZIK18*) were strongly down-regulated, while one gene (*ScRAF43*) had remained unchanged across all the time, as compared to the control treatment (0hpt) in ZT3 sugarcane cultivar (Fig. 8C). Significance differences and confidence intervals of MAPK cascade gene expression differences for each treatment time point are highlighted in supplementary Table S6.

## Discussion

### Plants possess extensive genetic diversity within the MAPK cascades gene families

A multitude of physio-biochemical activities are controlled by the highly conserved signal transduction system known as the MAP kinase cascade in plants [1, 31]. Recent genome-wide analyses of MAPK cascade gene families in various plant species have provided valuable insights into the mechanisms regulating MAP kinase modules and formed an important foundation for further functional characterization [2, 32, 33]. There is a wide range of variation in the number of members of the MAPK cascade family genes among the various plant species. For instance, 16 *ShMAPKKK*, Six *ShMAPKK*, 16 *ShMAPK* genes in sugarcane [2], 72 *hzsMAPKKK*, 10 *hzsMAPKK*, 24 *hzsMAPK* in maize inbred line Huangzaosi [4], 166 *GhMAPKKK*, 23 *GhMAPKK*, 52 *GhMAPK* in *Gossypium hirsutum* [34], 56 *FtMAPKKK*, one *FtMAPKK*, eight *FtMAPK* in *Fagopyrum tataricum* [6], and 152 *NnMAPKKK*, 15 *NnMAPKK* and 31 *NnMAPK* in *Nelumbo* [35]. In this study, 107 *ScMAPKKK*, 24 *ScMAPKK* and 89 *ScMAPK* genes were identified in the polyploid R570 genome, indicating the considerable genetic diversity among several plant species. Since the MAPK cascade genes were not clustered by monocots or dicots, phylogenetic analysis suggested that these genes likely expanded before monocots and dicots split. Similar phylogenetic classifications of MAP kinase cascade genes have been documented in *Saccharum* spp [2]., *G. hirsutum* [34], *F. tataricum* [6], *Nelumbo* [35], *Z. mays* [36, 37], *Triticeae* species [38], and *O. sativa* [39].

The two catalytic domains, STKc and PKc motifs, are associated with serine/threonine-kinases, not specifically threonine-kinases, and catalyze the phosphorylation of both serine and threonine amino acid residues within target proteins and are crucial for various function in most cellular activities [40]. Prior research has demonstrated that protein kinases and phosphoprotein phosphatases induce reversible phosphorylation through all MAPKs containing STKc and PKc motifs [1]. Additionally, MAP kinases belong to multi-gene families categorized by the occurrence of a T-loop with TEY, TDY, or MEY conserved motifs [2, 41]. The reported literature suggests that proteins with these motifs in their N-terminal regions might have a common evolutionary ancestor, or they may be phosphorylation sites for unknown kinases [42, 43]. In this study, 168 MAPK cascade genes (84 *ScMAPKs*, and 84 *ScMAPKKKs*) possessed the STKc domain, 52 genes (five *ScMAPKs*, 24 *ScMAPKKs*, and 23 *ScMAPKKKs*) had the PKc domain, and *ScMAPK* genes possessed an N-terminal TEY-/TDY-/MEY conserved motif. Additionally, members of the *ScMAPKKs* exhibiting D-(L/I/V/M)-K motif (active-site) and the S/T-xxxxx-S/T motif (T-loop) as well as other conserved subdomain motifs, for example, VGT-2x-YM-x-PER, D/I-3x-G/L, and FPY, previously identified in sugarcane cultivar R570 [2]. The *ScMAPKKK* genes possess a ‘MAP-E/V’ motif that constitutes the core aa residues of a kinase domain for three subfamilies of MAPKKKs (RAF-like, MEKK-like, and ZIK-like), which are identical to those found in bermudagrass [44], sugarcane [2], and tea plants [45].

The gene structure analysis showed that *ScMAPK*, *ScMAPKK*, and *ScMAPKKK* genes exhibit similar exon-intron arrangements within their phylogenetic groups. *ScMAPK* genes have variable exon and intron counts across groups, *ScMAPKK* genes range from simpler structures in Group-I to more complex forms in other groups, and *ScMAPKKK* genes display the highest structural diversity, especially within the MEKK-like and RAF-like subfamilies. The data suggest that MAPK cascade genes across different plant species exhibit similar gene structures and conserved evolutionary origins, implying that homologous genes likely share comparable biological functions. Furthermore, genes that belong to the same evolutionary group most likely play similar roles as they govern biological processes. Significant alterations in the number of introns and exons found within the *ScMAPKKK* gene family suggest that these genes in the *Saccharum.* spp. R570 genome has seen considerable diversity throughout genomic evolution. Previous studies in other plant species, including monoploid *Saccharum* spp. Hybrid R570 cultivar [2], *G. hirsutum* [34], *F. tataricum* [6], *Pyrus x bretschneideri* [46], *Nelumbo* [35], and *Corchorus* species [47] support our findings. Plant genome evolution is largely shaped by gene duplication, chromosomal segment duplication, and full genome duplication [48, 49]. Our findings showed that the polyploid *Saccharum* spp. Hybrid R570 genome had frequent gene duplication events of MAPK cascade genes, although no tandem duplications within the same chromosome were found. These findings point to the importance of chromosomal segment transposition and duplication events in the evolution of these gene families in sugarcane. Tandem duplication events are rare occurrences in the growth of the MAP kinase cascade gene families, according to earlier research [2, 8, 15, 45, 50].

Gene function and regulation are largely determined by *cis*-regulatory elements in the promoter region [51]. Our results suggest that the MAPK cascade genes in *Saccharum* spp. Hybrid R570 contained many MYB, MYC, and MBS *cis*-elements that are involved in drought responses [52]. Other stress-responsive elements were also identified in the three gene families, including the DRE core or DRE1 element, which is important for the responses to dehydration [53], the W-box that is involved in biotic stress [54], and LTR and STRE elements that are crucial for responses to low temperature and osmotic and oxidative stress, respectively [55]. Additionally, many *cis*-elements are involved in hormone responses, such as SA-responsive elements (TCA-element) and MeJA-responsive elements (CGTCA-motif and TGACG-motif), which are associated with the response to pathogen stimulus [56, 57]. Furthermore, various cis-elements play crucial roles in plant growth and development, such as circadian, RY-element, MSA-like, CAT-box, and HD-ZIP 1, which were found to be involved in zein metabolism, circadian control, seed-specific regulation, cell cycle activity, meristem expression, and differentiation of palisade mesophyll cells respectively [58]. Our findings suggest that diverse *cis*-regulatory elements involved in various cellular processes are present in the promoter regions of MAP kinase family members, likely acting as diverse functions related to growth, development, and stress response.

Gene duplication is an evolutionary mechanism that generates additional copies of genes, allowing them to evolve new roles or specialize existing ones. This process preserves essential biological processes while allowing mutations without immediate negative consequences. Regulatory divergence can result in differential gene expression, increasing genetic complexity, innovation, and adaptability, driving the evolution of new traits and biological pathways [59, 60]. The duplication of individual genes, chromosomal segments, and even entire genomes significantly influences genome structure and content during plant evolution [24]. Our data revealed that gene duplication events of MAPK cascade genes were frequently observed in the *Saccharum* spp. Hybrid R570 genome. However, no tandem duplication was detected within the same chromosome, suggesting that duplication events and chromosomal segment transposition may play crucial roles in the expansion of these genes during sugarcane genome evolution. Consistent with our findings, previous studies have also reported that tandem duplications are rare in the expansion of the *MAPK*, *MAPKK*, and *MAPKKK* gene families [1, 6, 11, 13].

MicroRNAs (miRNAs) are a type of small non-coding RNAs that control the expression of targeted genes by means of post-transcriptional processes, including translational inhibition or miRNA cleavage. They perform crucial functions in the development of plants as well as the development of defensive mechanisms in response to environmental stimulus [10, 61]. In this study, 13 different miRNAs were predicted to target 89 *ScMAPKs*, 24 *ScMAPKKs*, and 107 *ScMAPKKKs*. For instance, 87 genes (including 47 *ScMAPKs*, 22 *ScMEKKs*, and 18 *ScZIKs*) were targeted by 13 unique miRNAs from nine families, with ‘ssp-miR168a’ targeting the highest number of 45 genes. Specific miRNAs, such as ssp-miR444 and ssp-miR528, showed selective targeting of the gene members from MEKK-like, RAF-like, and ZIK-like subgroups. It indicates that these genes are likely controlled by sof-miR167, ssp-miR444a, ssp-miR444b.1, and ssp-miR444b.2, which might play as a critical role in biological processes via miRNA-MAP kinase modules implicated in stress responses in sugarcane. A previous study in rice disclosed that several miRNAs, such as OsmiR156, OsmiR159, OsmiR160, OsmiR166, OsmiR169, OsmiR171, OsmiR-172, OsmiR-199, OsmiR-393, OsmiR396, OsmiR397, OsmiR-408, and OsmiR444 have been reported as responsive miRNAs under heavy metal and heat or cold stress [62]. NtmiR-166, NtmiR167a, and NtmiR-169 were essential for controlling plant development and resistance to starvation of inorganic phosphorus (Pi) in *N. tabacum* [63]. Likewise, in maize, Zm-miR167 governs the expression of polyamine oxidase 1 (*ZmPAO1*), which subsequently modulates H_2_O_2_ generation, hence augmenting resistance to maize chlorotic mottle virus [64].

Previous comprehensive studies in plants have revealed that MAPK cascades play a crucial role in controlling a number of biological processes, including plant growth and development and responses to various biotic and abiotic stresses such as salt, cold and drought stress [65–67]. In our study, ScMAPKK20 (homologous to A0A1D6MU28), ScMAPKK12 (homologous to Mek1), ScMAPKK03 (homologous to Mek1), ScMAPK01 (homologous to Simk1), ScMAPK40 (homologous to MPK7) and ScMEKK20 (homologous to A0A1D6NHP1) proteins formed the core of this network. These proteins interacted with each other as well as with additional members of the MAPK cascade. These homologous proteins were considered STRING proteins because of the fact that proteins possessing high sequence and structural similarity tend to have similar functions. Therefore, we speculated that these proteins might have stronger interactions with other MAPKKK–MAPKK-MAPK proteins and play a key role in responding to abiotic stress and growth and development processes in plants. Previous studies have shown that the MEKK1-MKK1-MPK4 cascade is activated in response to wounding stress [68], while MEKK1-MKK2-MPK4/MPK6 cascade is stimulated in salt and cold stress conditions [69]. In *A. thaliana*, the MEKK1-MKK2-MPK4 signaling module is activated under cold stress and positively regulates cold response and freezing tolerance, whereas the MAPKKK YDA-mediated pathway negatively regulates MPK3/MPK6 activation in response to cold stress [70]. Additionally, the MEKK1 MKK4/MKK5-MPK3/MPK6 cascade is involved in plant defense against pathogen infections [71, 72]. The *MKK3* gene encodes a MAP Kinase Kinase, that activates *MPK8*, and is targeted by *MPKKK20*, thereby regulating ROS accumulation [73]. In rice, the OsMKKK10-OsMKK4-OsMAPK6 cascade participated in the regulation of grain size and weight [74]. Collectively, our interaction network analysis highlights the critical role of MAPKKK-MAPKK-MAPK signaling in stress responses. The identification of novel interaction pairs in sugarcane underscores the need for further investigation into MAPK cascade-mediated signaling pathways, which could provide valuable insights for genetic and functional studies.

The MAPK signaling cascades are pivotal in the complex molecular machinery governing plant immunity, orchestrating a complex defense response against diverse stresses [2]. This study examined the expression profile of six MAP Kinase genes in response to drought, salinity and calcium chloride stresses. Results show that these genes display distinct stress responses: *ScMAPK07*, *ScMAPK66*, and *ScRAF43* were down-regulated under drought, while *ScMAPKK13*, *ScRAF10*, and *ScZIK18* were up-regulated, with *ScZIK18* showed particular promise as a defense-related gene under drought condition. Under salinity (NaCl) stress, most genes were down-regulated except for slight increases in *ScZIK18* and *ScMAPKK13*. These expression patterns suggest that while salinity suppresses the expression of many signaling components, *ScZIK18* and *ScMAPKK13* may play specific roles in stress signaling and cellular defense, potentially enhancing sugarcane ability to regulate ion homeostasis and mitigate salt-induces damage. The findings are aligned with the earlier studies. For example, in rice, the *OsMAPKKK34* (homolog to *ScRAF43*) gene from Raf-subfamily, was up-regulated in response to both drought and salinity stress [75]. Added to that, *OsMAPKKK22* and *OsMAPKKK18* genes from MEKK-subfamily were activated by drought, whereas *OsMAPKKK20* (homolog to *ScZIK18*) gene from ZIK-subfamily was down-regulated in roots, but it responded to both drought and NaCl stressors in young leaves [75]. Similarly, in tomato plants, *SiMAPKK01*,* SiMAPKK02* and *SiMAPKK05* were up-regulated under heat, cold, and drought stresses. Notably, *SiMAPKK05* (homolog to *ScMAPKK13*) transcript was remarkably up-regulated by salt stress, while *SiMAPKK02* was strongly up-regulated by drought stress [76]. Furthermore, *SiMAPKKK6* is strongly up-regulated, and *SiMAPKKK40* (homolog to *ScRAF10*) show moderate upregulation under cold, heat, drought, and salinity stress across various time points [76]. In maize, *ZmMAPKKK56* expression was increased in the leaves and roots but declined in the stem during drought stress [77]. The *HaMKK3* and *HaMKK6* (homolog to *ScMAPK07*) genes in sunflower plants were up-regulated in response to NaCl and PEG stresses but down-regulated by salicylic acid (SA) treatment [78]. Overexpression of *GhMPK3* from cotton enhances cold, drought, and salt stress in *Arabidopsis* [79]. Similarly, *StMAPKK5* positively regulates response to drought and salt stress in Potato crop [80]. MAPK signaling also integrates with hormonal pathways to mediate stress responses. For example, *AtMAP3K17* and *AtMAP3K18* genes are regulated by the ABA core signaling module, activating C group MAPKs by MKK3, potentially triggering ABA-dependent responses in drought signaling in *Arabidopsis* [81, 82]. In foxtail millet, *SiMPK4/6*, which belongs to the same subfamily as *AtMAPK17/18*, responded positively to ABA and 10% PEG stress and was up-regulated [83]. In addition, *MAPKKK28* functions upstream of the MKK1-MPK1 cascade to regulate abscisic acid responses in rice [84]. Therefore, we hypothesize that MAPK cascade genes may be in a cross-talk between hormones and abiotic stress. Further research has uncovered the regulatory role of the *CaMEKK17* in interacting with *CaAITP1* and *CaAIPP1*, inhibiting its kinase activity, and functioning downstream of *CaAITP1* in ABA-mediated drought tolerance [85]. In sorghum, *SbMPK14* functions as a negative regulator of the drought response by suppressing the activity of specific ERF and WRKY Transcription factors [86]. In response to cold stress, calcium signaling acts as an early sensor, triggering MAPK cascades that regulate cold-responsive genes. This interaction ensures precise signal transduction, allowing plants to activate protective mechanisms. The cross-talk between calcium signaling and MAPK pathways fine-tunes gene expression, optimizing stress adaptation. Together, they enhance plant resilience and survival under cold conditions. In our study, calcium (CaCl₂) treatment in the ZT3 sugarcane cultivar led to strong down-regulation of most genes, while *ScRAF43* remained unchanged, suggesting that these genes may be negatively regulated by calcium stress, leading to enhanced cold sensitivity. This may be due to calcium-mediated signaling pathways selectively suppressing specific MAPK cascade components while maintaining basal expression of certain regulatory genes. This phenomenon has also been observed in other plants; for example, *MPK3* and *MPK6* are well-known conserved MAPKs, that inhibit cold tolerance in plants by facilitating the degradation of the ICE1 protein, whereas *CRLK1* and *CRLK2* reduce the activation of *MPK3* and *MPK6* during stress response, particularly cold stress [70]. However, the precise mechanisms of Ca^2+^-mediated MAPK signal transduction remain unclear. One reasonable hypothesis is that CRLKs regulate *MEKK1*-*MKK1*/*2*-*MPK4* cascade, where *MEKK1* is phosphorylated by *CRLK1* [87]. This cascade, in turn, negatively regulates *MPK4*-*MPK6* pathway [88]. More recently, Wang et al. [89] highlighted that *ZjMAPKK4* interacted with *ZjNAC78*, and VIGS-induced *ZjNAC78* silenced jujube plants showed cold sensitivity and the expression level of cold response genes were down-regulated after cold stress. These findings demonstrated that *ZjMAPKK4* could interact with *ZjNAC78* to regulate the downstream *ZjICE*-*ZjCBF* genes to regulate the cold tolerance of jujube [89]. As discussed earlier, Ca^2+^ signaling also positively influences MAPK-mediated plant response to cold stress. For instance, CoMAPKKK5/CoMAPKKK43/CoMAPKKK49-CoMAPKK4-CoMAPK8 module plays a key role in the cold stress resistance of wild *C. oleifera* [90]. Overall, these findings reveal the conserved roles of MAPK signaling in plant responses to abiotic stresses and underscore specific genes as potential targets for improving crop stress resilience.

## Conclusion

This study presents a genome-wide identification of MAP Kinase cascade genes in the polyploid *Saccharum* spp. Hybrid R570, revealing 89 *ScMAPKs*, 24 *ScMAPKKs*, and 107 *ScMAPKKKs*. The characteristics of these MAPK cascade genes showed that they had a high structural diversity and an abundance of *cis*-elements linked to stress responses (biotic and abiotic), suggesting their functional diversity in sugarcane adaptation to environmental stresses. Additionally, 87 MAPK cascade genes were targeted by various miRNAs, such as “sof-miR167, ssp-miR444a, ssp-miR444b.1, and ssp-miR444b.2”, highlighting a role for post-transcriptional regulation in stress responses via the MAP Kinase signaling pathway. qRT-PCR results further revealed diverse expression patterns of MAP Kinase cascade family members under PEG, NaCl and CaCl_2_ stressors, implying that these may participate in multiple signaling pathways and cross-talk within various MAPK modules. The precise role of these genes in sugarcane adaptation to abiotic stresses necessitates additional investigation. This study provides a thorough examination of the *Saccharum* spp. MAPK cascade gene families, improving our comprehension of the roles of *ScMAPKs*, *ScMAPKKs*, and *ScMAPKKKs* in sugarcane.

## Electronic supplementary material

Below is the link to the electronic supplementary material.

Supplementary Material 1

Supplementary Material 2

Supplementary Material 3

Supplementary Material 4

Supplementary Material 5

Supplementary Material 6

Supplementary Material 7

Supplementary Material 8

Supplementary Material 9

Supplementary Material 10

## Data Availability

No datasets were generated or analysed during the current study.
