# Supplementary figures and images for: Genome-wide identification and unveiling the role of MAP kinase cascade genes involved in sugarcane response to abiotic stressors
Source: BMC Plant Biol. 2025 Apr 16;25:484. doi: 10.1186/s12870-025-06490-1 (PMC12001561; doi:10.1186/s12870-025-06490-1)

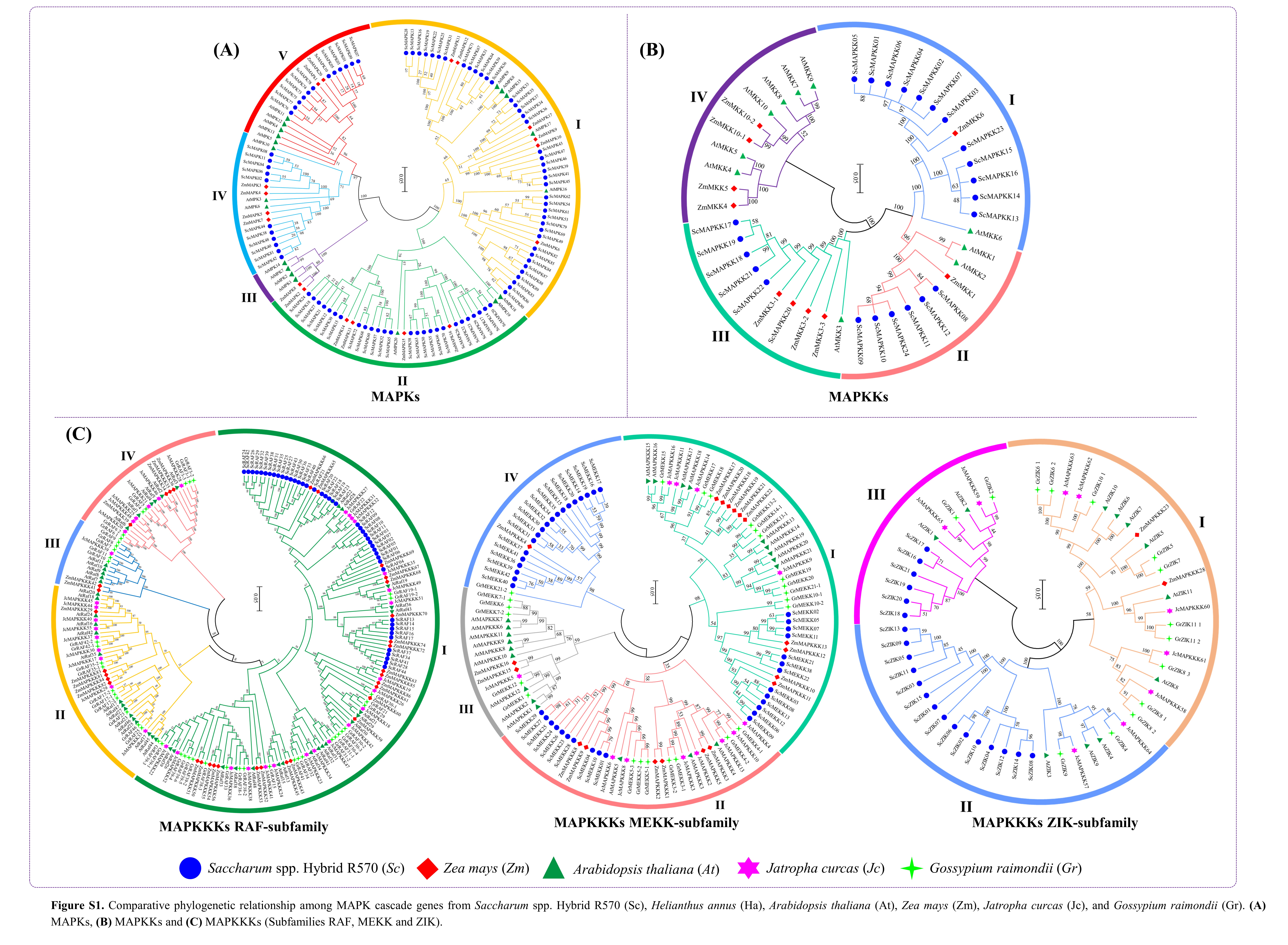

Supplement: Supplementary file 1 — Supplementary Material 1 [file 12870_2025_6490_MOESM1_ESM.jpg]

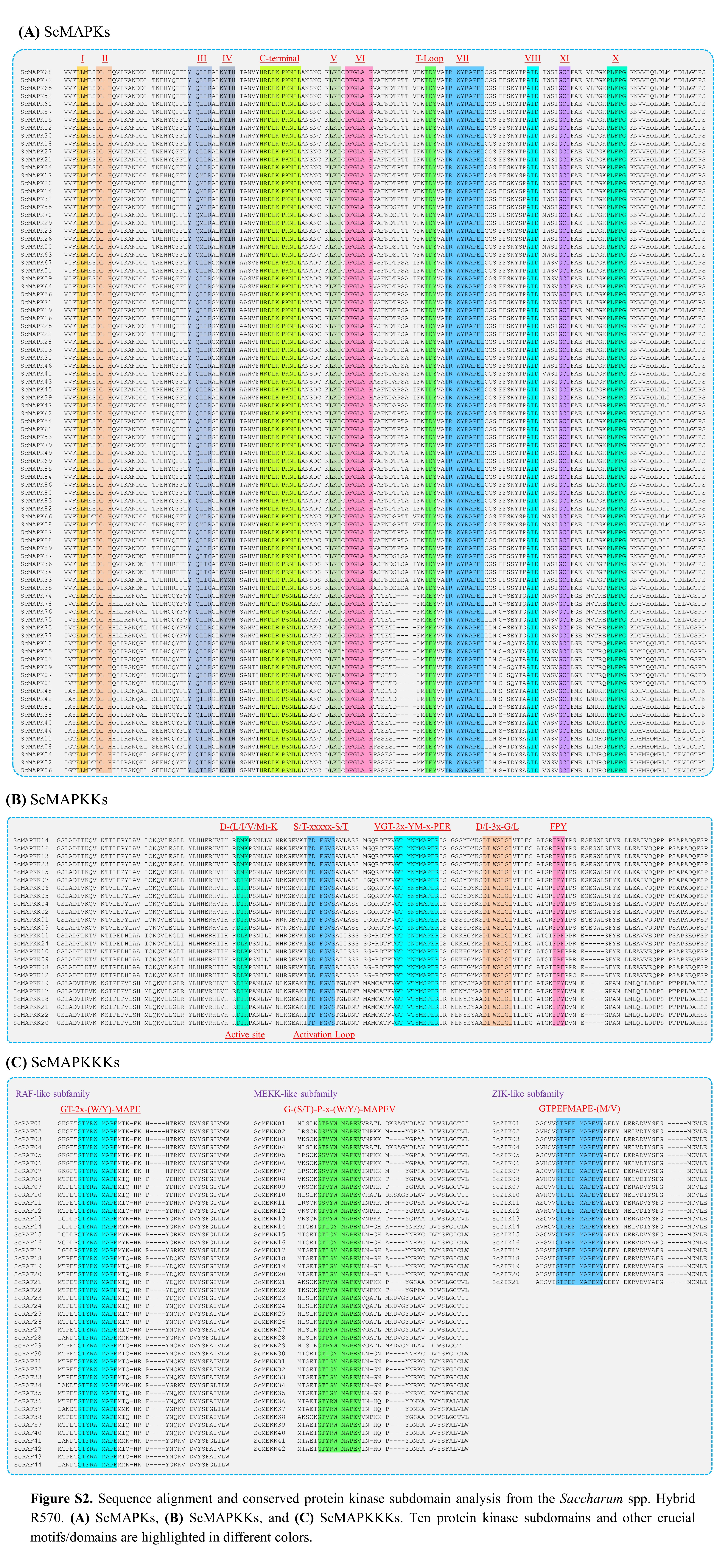

Supplement: Supplementary file 2 — Supplementary Material 2 [file 12870_2025_6490_MOESM2_ESM.jpg]

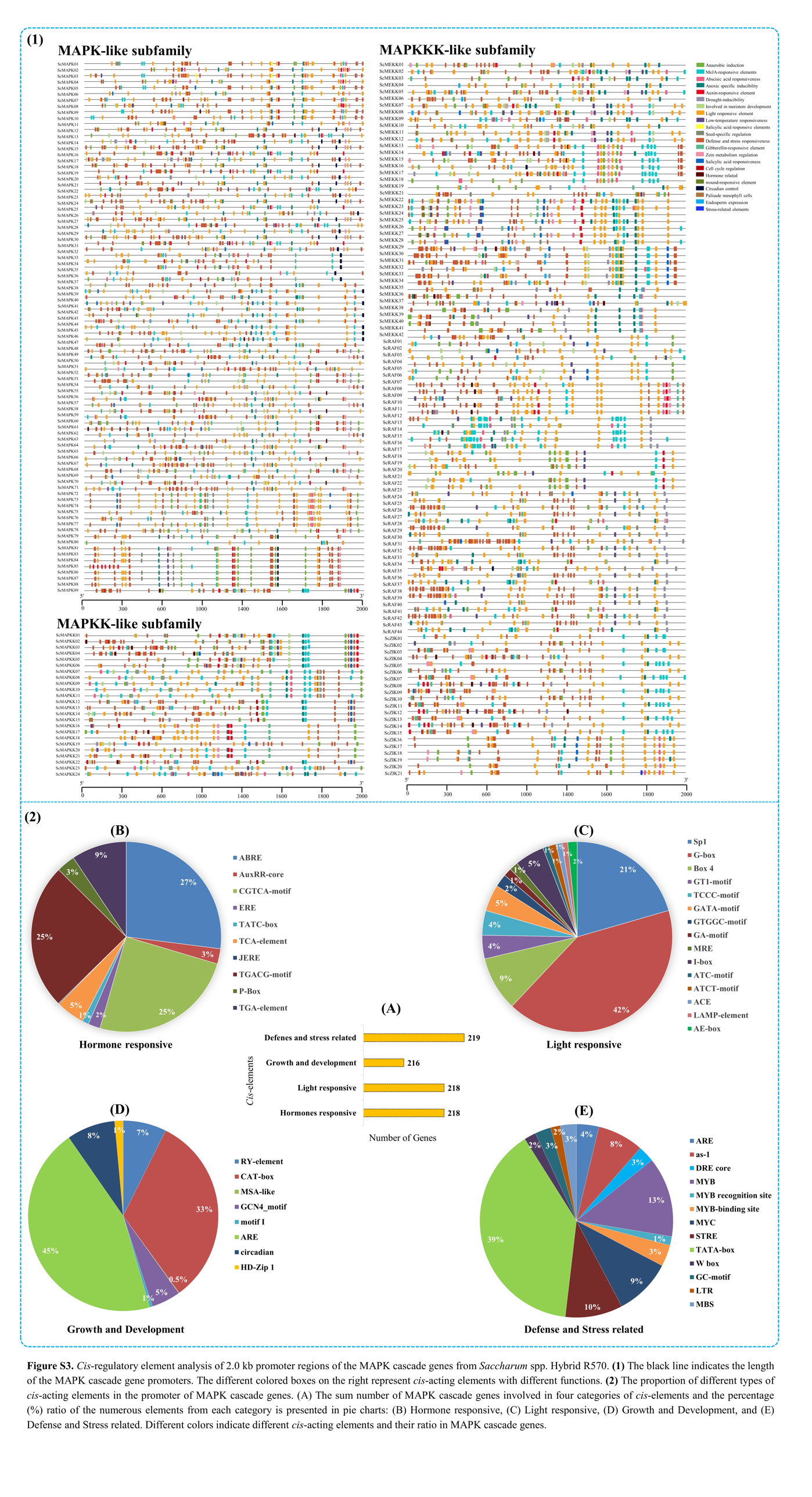

Supplement: Supplementary file 3 — Supplementary Material 3 [file 12870_2025_6490_MOESM3_ESM.jpg]

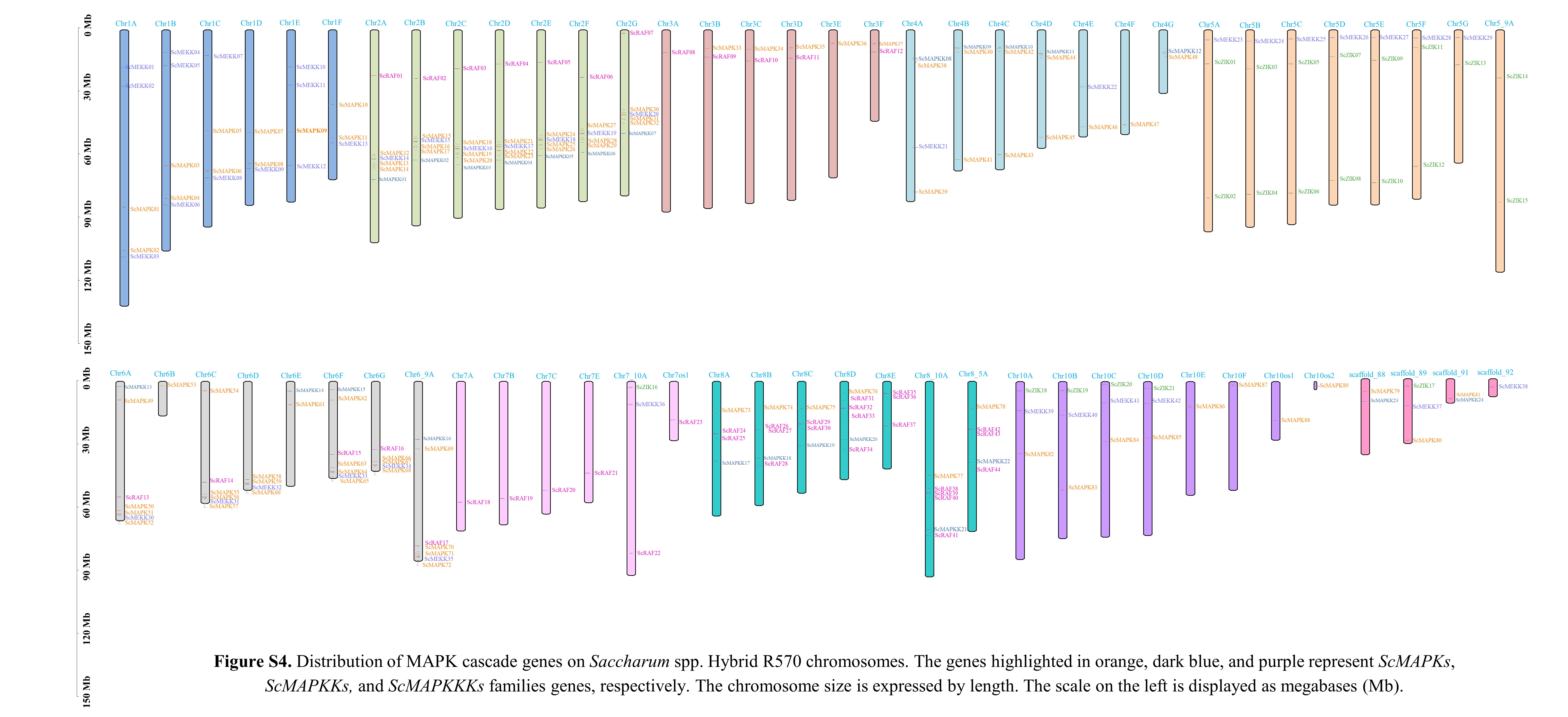

Supplement: Supplementary file 4 — Supplementary Material 4 [file 12870_2025_6490_MOESM4_ESM.jpg]
